# Validation of the fermented food frequency questionnaire to assess consumption across four European regions: a study within the promoting innovation of fermented foods cost action

**DOI:** 10.3389/fnut.2025.1667653

**Published:** 2025-10-29

**Authors:** Emmanuella Magriplis, Theodoros Smiliotopoulos, Niki Myrintzou, Kathryn Jane Burton-Pimentel, Signe Adamberg, Kaarel Adamberg, Duygu Agagündüz, Natalijam Atanasova-Pancevska, Meline Beglaryan, Elske M. Brouwer Brolsma, Johana Burtscher, Marija Cerjak, Zuzana Ciesarová, Inga Ciprovica, Francesca De Filippis, Mónica Gandía, Elaine Hillesheim, Luziana Hoxha, Daniel Borch Ibsen, Nastia Ivanova, Håvard Jenssen, Petra Jones, Anastasia Kalea, Zeynep Begum Kalyoncu Atasoy, Vaida Kitryte-Syrpa, Aleksandar Kostic, Marta Laranjo, Victoria Meslier, Nóra Emilia Nagybákay, Gjore Nakov, Malviina Nikola, Christos Pafilas, Photis Papademas, Foteini Pavli, Tagli Pitsi, Terhi Pohjanheimo, Igor Pravst, Jelena Rajic, Pasquale Russo, Taner Sar, Małgorzata Starowicz, Irzada Taljic, Biljana Trajkovska, Guy Vergères, Bojana Vidovic, Christophe Chassard, Michail Syrpas

**Affiliations:** ^1^Laboratory of Dietetics & Quality of Life, Department of Food Science and Human Nutrition, Agricultural University of Athens, Athens, Greece; ^2^Agroscope, Federal Office for Agriculture (FOAG), Federal Department of Economic Affairs, Education and Research (EAER), Bern, Switzerland; ^3^Department of Chemistry and Biotechnology, Tallinn University of Technology, Tallinn, Estonia; ^4^Department of Nutrition and Dietetics, Gazi University, Emek, Ankara, Türkiye; ^5^Department of Microbiology and Microbial Biotechnology, Faculty of Natural Sciences and Mathematics, Institute of Biology, Ss. Cyril and Methodius University in Skopje, Skopje, North Macedonia; ^6^Center for Ecological-Noosphere Studies, National Academy of Sciences of the Republic of Armenia (NAS RA), Yerevan, Armenia; ^7^Division of Human Nutrition and Health, Wageningen University & Research, Wageningen, Netherlands; ^8^BOKU University, Institute of Food Science, Department of Biotechnology and Food Science Vienna, Vienna, Austria; ^9^Department of Food Science and Technology, Institute of Food Science, Vienna, Austria; ^10^Faculty of Agriculture, University of Zagreb, Zagreb, Croatia; ^11^National Agricultural and Food Centre, Food Research Institute, Bratislava, Slovakia; ^12^Latvia University of Life Sciences and Technologies, Faculty of Food Technology, Jelgava, Latvia; ^13^Department of Agricultural Sciences, University of Naples Federico II, Portici, Italy; ^14^Food Technology Area, Preventive Medicine and Public Health, Food Science, Toxicology and Forensic Medicine Department, Faculty of Pharmacy and Food Sciences, University of Valencia, Valencia, Spain; ^15^Faculty of Biotechnology and Food, Agriculture University of Tirana, Tiranë, Albania; ^16^Department of Public Health, Aarhus University, Aarhus, Denmark; ^17^College of Sliven, Technical University of Sofia, Sliven, Bulgaria; ^18^Department of Chemistry, University of Oslo, Oslo, Norway; ^19^Department of Science and Environment, Roskilde University, Roskilde, Denmark; ^20^Department of Food Sciences and Nutrition, Faculty of Health Sciences, University of Malta, Msida, Malta; ^21^Division of Medicine, University College London, London, United Kingdom; ^22^Department of Nutrition and Dietetics, Gulhane Health Sciences Faculty, University of Health Sciences, Ankara, Türkiye; ^23^Department of Food Science and Technology, Kaunas University of Technology, Kaunas, Lithuania; ^24^Department of Food Technology and Biochemistry, Faculty of Agriculture, University of Belgrade, Belgrade, Serbia; ^25^MED-Mediterranean Institute for Agriculture, Environment and Development & CHANGE-Global Change and Sustainability Institute, Departamento de Medicina Veterinária, Escola de Ciências e Tecnologia, Universidade de Évora, Évora, Portugal; ^26^MetaGenoPolis, Institut National de Recherche pour l'Agriculture, l'Alimentation et l'Environnement, University Paris-Saclay, Jouy-en-Josas, France; ^27^Aistila Ltd., Turku, Finland; ^28^Department of Agricultural Sciences, Biotechnology and Food Science, Cyprus University of Technology, Limassol, Cyprus; ^29^Department of Nutrition and Exercise, The National Institute for Health Development, Tallinn, Estonia; ^30^Biotechnical Faculty, University of Ljubljana, Ljubljana, Slovenia; ^31^Department of Bromatology, Faculty of Pharmacy, University of Belgrade, Belgrade, Serbia; ^32^Department of Food, Environmental and Nutritional Science (DeFENS), University of Milan, Milan, Italy; ^33^Swedish Centre for Resource Recovery, University of Borås, Borås, Sweden; ^34^Team of Chemistry and Biodynamics of Food, Institute of Animal Reproduction and Food Research, Olsztyn, Poland; ^35^Faculty of Agriculture and Food Sciences, University of Sarajevo, Sarajevo, Bosnia and Herzegovina; ^36^Faculty of Biotechnical Sciences, University St. Kliment Ohridski, Bitola, North Macedonia; ^37^UCA, INRAE UMRF, VetAgroSup, Aurillac, France

**Keywords:** fermented foods, dietary assessment, food frequency questionnaire, 24-h dietary recalls, validation, Bland-Altman analysis

## Abstract

**Background:**

Fermented foods are an integral part of diets worldwide, and emerging epidemiological data suggest their significant beneficial health effects. However, assessing their intake is challenging since many of these foods are sporadically and/or locally consumed, hence current traditional nutritional assessment tools lack the specificity to capture this variability. To address this gap, the Fermented Food Frequency Questionnaire (3FQ) was developed and this study aimed to evaluate its relative validity and repeatability across European regions.

**Methods:**

In the validation study of the 3FQ, 12,646 adult participants were recruited across four European regions to assess consumption of sixteen major fermented food groups. Repeatability was assessed by administering the 3FQ twice, ~6 weeks apart, to a subset of participants (*n* = 2,315). Validity was evaluated using 24-h dietary recalls (24 h). Statistical analyses included Spearman's rank correlation coefficients and Intra-Class Correlation coefficients (ICC) for repeatability, and Bland-Altman plots for validity.

**Results:**

Results showed high repeatability, overall and by region, for estimated quantities and frequencies of consumption for most of the fermented food groups (from 0.4 to 1.0), with a few exceptions for infrequently consumed items (e.g., fermented fish). Validity assessment via Bland-Altman plots revealed excellent agreement between the 3FQ and 24 h for most of the food groups, with over 90% of values falling within the agreement interval. Notably, fermented dairy products, coffee, and bread categories showed the strongest agreement (>95%).

**Conclusion:**

The 3FQ is a robust and reliable tool for estimating the consumption of diverse fermented food groups across four European regions and provides valid estimates of the frequency and quantity of intake for specific fermented foods. The 3FQ could be a valuable instrument for epidemiological research aiming to elucidate associations between certain fermented foods and health parameters in European diets.

## Introduction

Fermented foods are an integral part of diets worldwide, with strong cultural and regional associations (1, 2). The importance of identifying fermented food consumption patterns is increasingly recognized with new epidemiological data regarding their potential broad health effects (3–5). Standardized dietary assessment tools are fundamental for accurately capturing population-level dietary intake data in epidemiological studies (6); however, unlike most staple foods, fermented food intake is challenging to accurately capture, since they are either occasionally and/or locally consumed. In fact, there are only a limited number of studies that have attempted to define fermented foods and assess their intake prevalence, and contribution to energy and nutrients, as they are often consumed sporadically, and are often included as side dishes hence being “forgotten” (7–9). Moreover, the type of fermented food and the amounts consumed vary considerably between populations; a reliable nutritional tool is required to consistently capture this diversity in intake (6).

Food Frequency Questionnaires (FFQs) are practical tools that can be adapted to assess diversity in food consumption and dietary habits over extended periods (6, 10). However, they are subject to several methodological challenges that must be addressed to ensure the validity and reliability of dietary intake assessments. These limitations include recall bias, portion size estimation inaccuracies, and variability due to participants' cognitive status and educational level (11). These can be mitigated by using well-structured, straightforward questions, with clear food images, improving usability and response accuracy by individuals with diverse educational backgrounds and cognitive abilities (6, 10). In the development of a *de novo* FFQ, it is essential to start with previously validated dietary assessment tools that are closely aligned with the target population and the specific food groups of interest. These serve as the basis for constructing tailored FFQs, often referred to as screeners, that effectively capture culturally relevant food items and appropriate portion sizes (11). To enhance, however, the tool's efficiency and reliability in assessing participants' actual intakes, it must undergo validation in terms of repeatability and validity through comparison with a gold-standard dietary assessment method (12, 13). An efficient validation approach involves comparing FFQ data with repeated 24 h recalls (24 h), serve as a reliable reference method due to their ability to provide detailed, short-term intake data (12, 13). In multi-country epidemiological studies, these validation approaches enhance cross-population comparability by capturing regional dietary differences and improving the adaptability of the FFQ across diverse dietary patterns (11). Additionally, assessing repeatability, a psychometric property reflecting the tool's ability to yield consistent results under similar conditions, is crucial to demonstrate stability over time and minimize random error, thereby ensuring questionnaire reliability (14, 15).

Incorporating biomarkers into dietary assessments could improve the validity of the reported dietary intake by reducing reporting bias. However, biomarkers must be validated for reproducibility, sensitivity, and applicability to the specific population before use in nutritional studies (6). Additionally, epidemiological studies lack identified and validated biomarkers for detecting the consumption of specific, yet diverse food categories such as fermented foods (16, 17). Fermented foods have gained significant attention for their potential health benefits, as cited by various scientific studies (4, 5), although these potentially vary according to their diversity. There is a need, therefore, to quantify the habitual consumption of fermented foods across the whole diet and by food group. To our knowledge, no validated tool has been developed to date for assessing the habitual consumption of diverse fermented foods.

To address this gap, a novel FFQ specifically designed to capture the consumption patterns of fermented foods across the main food groups was developed: the Fermented Food Frequency Questionnaire (3FQ) (18). This study aimed to examine the relative validity including accuracy and repeatability of the 3FQ, and to assess population intake of specific fermented foods grouped into 16 major food groups, and some frequently consumed subgroups, across European regions. This work is part of the Promoting Innovation of ferMENTed fOods (PIMENTO) COST Action (CA20128) (19).

## Methods

### Study design and population

The validation study was conducted between 2023 and 2024 as part of the PIMENTO COST Action (CA20128). Participants were adults over 18 years, living in one of the four European regions. Specifically *Northern Europe*: Estonia, Finland, Faroes, Iceland, Latvia, Lithuania, Norway, Denmark, Sweden; *Southern Europe*: Gibraltar, Greece, Italy, Malta, Portugal, San Marino, Türkiye, Holy See, Cyprus, Spain; *Central and Eastern Europe*: Albania, Armenia, Azerbaijan, Georgia, Hungary, Poland, Romania, Bulgaria, Belarus, Bosnia and Herzegovina, Croatia, Kosovo, North Macedonia, Montenegro, Moldova, Slovakia, Czechia, Russia, Serbia, Slovenia, Ukraine, Serbia and Montenegro; and *Western Europe*: Austria, Belgium, France, Germany, Ireland, Liechtenstein, Luxembourg, Andorra, Monaco, Netherlands, United Kingdom, Switzerland as defined by the EuroVoc (20). The questionnaire was disseminated online through the PIMENTO website, social media, email-based invitations of past survey participants from mailing lists (convenience sampling), and non-probability snowballing methodology. Respondents were encouraged to share the invitation with interested persons to achieve a more diverse audience. Ethical approval for this study was received from the Ethics Committee of the Agricultural University of Athens (27/05.05.2023) to certify compliance with research standards with human participants. Only one of the principal investigators (PI) had access to personal data shared by volunteers, who agreed to be recontacted (through phone call or email). All other personal identifications were pseudonymised until all analysis were completed and then anonymized in the final database. Details of the study design have been published in the study protocol (18).

### Development of the 3FQ

The Fermented Foods Frequency Questionnaire (3FQ) was developed in English with the collaboration of a group of food scientists, dietitians and epidemiologists (18). The questionnaire was designed to assess the consumption of fermented foods across an array of food groups (16 major with subgroups within each group as shown in Table 1), comprising dairy products, legumes, meat and fish products, plant-based alternatives, vegetables, chocolate, alcoholic and non-alcoholic beverages, and condiments. Each main food group presented in Table 1 represents the summation of the respective subgroups reported in the 3FQ and was calculated post 3FQ's data collection. For example, food group “vinegar” comprises the total consumption of the following categories: “Apple vinegar,” “Grape vinegar,” “Balsamic vinegar” and “Other types of vinegar (e.g., rice vinegar, white vinegar, etc.).” Validated food pictures were used to help participants identify the amount usually consumed and to ensure consistency in reporting, decreasing measurement error. Also, participants were asked to define the frequency of consumption for each food group, selecting from predefined continuous intervals (Never, Rarely, Monthly 1–2 times, Weekly 1–2 times, Weekly 3–6 times, Daily one time, Daily two times and Daily more than two times). For the quantification process, validated food pictures were carefully chosen following discussion with the NCP's and available National health surveys, to reflect low and large portion sizes for each subgroup included. A detailed database per picture used (food atlas book reference, page number, and allocated grams) with each picture referring to specific portion intake (weights) as detailed in each food atlas used. In the same database volumes of glasses and cups used as household measures were also included. Individuals were asked to selects the usual portion consumed each time.

**Table 1:** Foods and beverages included in the fermented food frequency questionnaire (3FQ).

**Fermented products (main) groups and subgroups**	
**Plant-based meat and/or dairy**	**Dairy products**
**alternatives**	**(animal-based)**
Plant-based cheese	Hard cheese (i.e., Parmigiano Reggiano, Grana Padano)
Plant-based meat	Semihard cheese (i.e., Edam, Gouda)
Plant-based yogurt	Semihard cheese (i.e., Gorgonzola, blue cheese)
Fermented pulses and legumes	Soft and/or fresh cheese (i.e., feta, quark)
Fermented chickpeas (e.g., dosa, dhokla, etc.)	Soft and/or fresh cheese (i.e., goat cheese, Camembert, Brie)
Fermented beans (e.g., miso, douchi, etc.)	Yogurt (i.e., greek-style, drinkable yogurt, Skyr, Kvarg)
Fermented pulse or legume-based sauces (e.g., soy sauce, tamari, etc.)	
**Fermented vegetable products**	**Fermented meat and/or**
	**fish products**
Olives	Dry sausage/dry aged meat (e.g., salami, chorizo, cecina, etc.)
Cabbage (sauerkraut)	Fermented fish (e.g., surströmming)
Others (e.g., carrots, cucumber, beetroot etc.)	Fermented fish or meat-based sauces (fish sauce, oyster sauce, etc.)
**Vinegar**	**Chocolate**
Apple vinegar	Milk chocolate
Grape vinegar	Dark chocolate
Balsamic vinegar	White chocolate
Other (e.g., rice vinegar, white vinegar, etc.)	
**Fermented cereal products**	**Fermented non-alcoholic**
	**beverage products**
White bread	Kombucha
White sourdough bread	Cereal drink (e.g., amazake, boza, braga, etc.)
Whole grain bread	Water kefir
Whole grain sourdough bread	
Other (tarhana/trahana, kishk, idli, etc.)	
**Coffee**	**Cocoa beverages**
Espresso (ristretto, lungo)	Sweetened
Filter coffee	Unsweetened
Arabic, Turkish, Greek, (boiled) coffee	
Instant (soluble) coffee	
**Fermented tea**	**Beer or cider**
Pu'er/puerh	Beer
Anhui Lu'an basket tea	Alcohol-free beer
Guangxi Liubao tea	Cider
Hubei green brick tea	Alcohol-free cider
Fu Zhuan	
Sichuan border tea	
**Wine (including sparkling)**	**Strong spirits**
White	Whisky/vodka/gin/brandy etc.
Red	
Rosé	
Alcohol free wine	

A standardized translation protocol was used to translate the 3FQ into multiple European languages. Native-speaking translators performed the initial translation and it was further translated into all national languages in countries that spoke more than one language (such as Switzerland, which translated into French, Italian, and German). Afterwards, blinded translators performed a back translation to identify discrepancies between the original and the translated questionnaire, which were reviewed and resolved to guarantee conceptual equivalence. Moreover, the cultural adaptation of the questionnaire was ensured by using different fermented food examples specific for each country while maintaining a consistent grouping system across all participating countries. For instance, in the cheese group, Italy used Mozzarella, and Greece used Feta, which are typical examples of soft or semi-soft cheeses in these countries.

### 3FQ validation methodology

To examine the 3FQ's repeatability, a set of participants who had consented to the process, were invited to complete the questionnaire a second time ~6 weeks following the first completion (within 4–8 weeks). Specifically, during the initial evaluation, they were asked whether they would be interested in participating in a follow-up assessment. Those who consented were asked for their email address or phone number for future communication. To ensure adequate data for regional comparisons and repeatability assessment per European Region, the study aimed to recruit a minimum of 200 people from each European region for the repeatability evaluation (see Statistical Analysis).

### 24-h dietary recalls

To validate the accuracy of the 3FQ, one or two 24-h phone or video-based recalls, using the Automated Multipass Method, from a representative sample of the study target population (21). A total of 218 individuals was required based on study power calculations for 90% power, assuming a low correlation of ρ_o_ = 0.4, and 265 to 371 individuals if the rule of thumb (five to seven participants per survey question). Sample size details are included in the protocol (18). Greece, Finland, Malta, Switzerland, Italy, Serbia, North Macedonia, Bulgaria, Latvia, Lithuania, Sweden, Estonia and Denmark were the countries where trained interviewers performed 24 h. To maintain the consistency of the data collected by the interviewers and improve the reliability of the 24 h process, standardized training was performed for all interviewers. An experienced dietitian delivered two online training sessions and an onsite workshop. Specific emphasis was placed on identifying and probing for fermented foods, given their diverse forms and varying levels of consumption across European populations. Interviewers were also trained to use appropriate questioning techniques to improve portion size estimation and minimize recall bias. To ensure uniformity in data collection from all countries, a database that provided all the required information to be extracted was created and used during the interviews by all participating countries.

The interviews were performed using the Automated Multipass Method (AMPM), a structured approach designed to enhance the accuracy and completeness of dietary data collection (22). This method was used to minimize underreporting and improve the report's overall accuracy. The initial step was for the participants to list all the foods and beverages they consumed the previous day. Following this, they were prompted for any forgotten foods through various methods, including reporting time and place of consumption for each meal. A detailed description of all the foods they consumed was requested, including portion sizes, preparation methods, and brand-specific information where applicable, while using specific grids, household volume measures (such as glasses, cups, plates, and various spoon sizes), and age-specific food atlases helped identify portion sizes. The EPIC food Atlas was used for countries that did not have their own. Before the end of the interview, a summary of the meals and drinks list was restated to confirm that the recall was complete and detect possible omissions. All selected portions were converted to grams of intake based on the detailed database available for each food atlas/picture book used.

To assess fermented food intake, single-ingredient foods and multi-ingredient preparations (recipes) were treated as separate items for quantification. Fermented food intake was assessed based on the reported amount consumed for foods containing only one ingredient. On the other hand, each recipe was broken down into its separate ingredients, and the contribution of each ingredient to the total weight was calculated, accounting for specific food and preparation yield factors. The percentage of fermented food consumed in the recipe was then applied to the total weight consumed as stated by the participant during the 24 R interview. This approach ensured a standardized quantification of fermented food consumption, enabling an accurate comparison between the 3FQ and the data from the 24 h.

### Statistical analysis

The required sample size for the validation study was determined through a power analysis using the G^*^Power application. Specifically, for the Spearman correlation analysis calculation to achieve 90% power, assuming a low correlation of ρ_o_ = 0.4 (the Null Hypothesis H_o_) with the potential of no correlation (the alternative Hypothesis H_1_ = 0.2), at alpha = 5%, two-tailed exact test, showed that 218 participants were required. Details on the process can be viewed in the published study protocol (18). The relative validity of the 3FQ was evaluated by contrasting estimates of fermented food consumption obtained from the questionnaire with those calculated from the average of two 24 h and using Bland-Altman agreement plots.

The distribution of continuous variables was evaluated using P-P plots and by assessing their standard deviation (SD) against their means (23). Based on the results of this data exploration, means and standard deviations (SD) were used to describe normally distributed continuous data, and medians (with 25th−75th percentiles) were used for skewed continuous data. All categorical variables were presented as percentages.

Several statistical techniques were employed to assess the repeatability of the 3FQ by examining the agreement within repeated assessments of the 3FQ. Spearman's rank correlation coefficients were used to determine the strength of associations between the two repetitions of the 3FQ (repeatability of the 3FQ). Spearman's correlation coefficient is interpreted as a weak correlation if rho = 0.1–0.3, a moderate correlation if rho = 0.3–0.5 and a strong correlation if rho = 0.5–1.0. Cross-classification analysis was conducted to establish the number of participants who were classified into the same or contiguous (i.e., neighboring) quartiles of intake in the questionnaire and the 24 h, clarifying the consistency of ranking in the methods used. Intra-class correlation coefficients (ICC) were also calculated to verify the robustness of Spearman's rho (24, 25); a method recommended for correlations within repeated measures of new experimental tools, considering the respective variances in order to assess the questionnaire's internal consistency and reliability (21, 26). It is interpreted as follows: ICC <0.5 poor; 0.5 ≤ ICC <0.75 moderate; 0.75 ≤ ICC <0.9 good; and ICC ≥0.9 excellent reliability. Weighted Cohen's kappa statistics was used to test interrater reliability for the broad questions (yes or no) that referred to the consumption of the main food groups used by the 3FQ. Cohen's Kappa statistic is interpreted as follows: values ≤ 0 indicates no agreement, 0.01–0.20 none to slight, 0.21–0.40 fair, 0.41–0.60 moderate, 0.61–0.80 substantial and 0.81–1.00 almost perfect agreement (27). Regarding the relative validity of the 3FQ, Bland-Altman plots were used to visually examine the scale of agreement and identify potential systematic bias in the reported intake. This method is generally accepted as a suitable tool for investigating differences between matched pairs derived from two different methods of measurement (24, 28). The test relies on constructing limits of agreement using the mean and standard deviation (SD) of the differences between the two measurements. If more than 90% of the data lie within ±2 SD, the results appear to agree (29). Spearman's rank correlations were also explored as a preliminary assessment of the accuracy-validity of the 3FQ using the cut-offs described above for the repeatability tests. Statistical analyses were performed using STATA MP 18 software (Texas ltd, Texas, USA). Statistical significance was set at alpha 5%.

## Results

The core study sample (1st 3FQ completion) consisted of 12,646 participants. The basic anthropometric and socio-demographic characteristics of the study population are presented in Supplementary Table 1. Shortly, The study sample had a mean age of 41 years, the majority held at least a university degree, and the mean Body Mass Index (BMI) was 24.5 kg/m^2^. Significant differences between the total sample and the sample that repeated the questionnaire were found in marital status, educational level, scientific background in nutrition. For nearly all the characteristics assessed by repetition of the 3FQ, the reliability of reporting was found to be very strong to almost perfect in the Spearman's correlation assessments.

Table 2 describes the repeatability of the consumer questions (yes-or-no type questions) that preceded each main food group's detailed food items in the 3FQ. The correlation between repetitions of the questionnaire ranged from moderate too strong for the 16 food groups assessed. Specifically, fermented: pulses and legumes, meat and/or fish, vegetables, cereals, and non-alcoholic beverage products, similarly, to fermented: tea, chocolate, vinegar, and coffee, were found to be moderately correlated among the two repetitions of the 3FQ. All the other food groups were found to be strongly correlated. Additionally, Cohen's kappa statistic ranged from moderate (“fermented pulses and legumes,” “fermented vegetable products,” “fermented cereals,” “chocolate,” and “fermented tea”) to almost perfect agreement between the two repetitions of the 3FQ.

**Table 2:** Spearman's correlation coefficients and Cohen's kappa statistic to check repeatability of the main 3FQ food groups by repetition of the 3FQ1 for the whole sample of participants (*n* = 1,247).

**Food group**	**1st 3FQ^a^**	**2nd 3FQ^a^**	**Spearman correlation coefficient^b^**	**Cohen's kappa statistic^c^**
Plant-based meat and/or dairy alternatives	55.24%	55.72%	0.632^**^	63.05^**^
Fermented dairy products (animal-based)	94.96%	95.44%	0.668^**^	66.74%^**^
Fermented pulses and legumes	68.29%	66.05%	0.532^**^	53.19%^**^
Fermented meat and/or fish products	73.82%	75.02%	0.607^**^	60.68%^**^
Fermented vegetable products	90.15%	91.03%	0.583^**^	58.20%^**^
Fermented cereal products	93.59%	94.72%	0.523^**^	52.02%^**^
Chocolate	93.03%	93.43%	0.588^**^	58.75%^**^
Fermented non-alcoholic beverage products	26.74%	26.50%	0.723^**^	72.33%^**^
Vinegar	73.10%	73.34%	0.675^**^	67.54%^**^
Coffee	81.59%	82.07%	0.865^**^	86.54%^**^
Fermented tea	41.87%	39.15%	0.542^**^	54.19%^**^
Cocoa beverages	48.12%	47.80%	0.692^**^	69.20%^**^
Beer or cider	63.17%	62.45%	0.837^**^	83.72%^**^
Wine	66.77%	66.61%	0.877^**^	87.75%^**^
Strong spirits	49.80%	48.36%	0.782^**^	78.22%^**^

^**^Correlation is significant at the 0.01 level (two-tailed).

3FQ, fermented food frequency questionnaire.

^a^n, study sample population.

^b^Spearman's correlation coefficient was used to determine the level of correlation between the two repetitions of the 3FQ.

^c^Weighted Cohen's kappa statistics was used to test interrater reliability for the broad questions (yes or no) that referred to the consumption of the main food groups used by the 3FQ.

The repeatability results for the estimated consumption (quantity and frequency) of each separate food group or food item described in the 3FQ are given in Tables 3, 4. Spearman correlation test results showed strong to very strong correlation between the estimated quantities and frequencies reported in the two repetitions of the 3FQ for all main food groups and individual food items, except for “fermented fish.” For this particular subgroup, although frequency results were found to be strongly correlated between repeated administrations of the 3FQ, quantity (as grams per day) showed only a weak non-significant association (rho = 0.272), due to insufficient amount of 3FQ answers for this particular food. The results for the ICC test that was used to assess interrater correlation for the reported quantities consumed were consistent with the Spearman's correlation test. Specifically, all 3FQ food groups and items showed moderate to excellent reliability for quantitative assessment of all main food groups and most food items except for “fermented chickpeas,” “fermented fish,” “white chocolate,” “cereal drinks,” “other types of vinegar,” “Guangxi tea” and “Hubei tea” that were found to be poorly reliable following the ICC test results.

**Table 3:** Spearman's correlation and ICC coefficients to assess repeatability of the quantification of 3FQ^a^ using the whole sample (grams per day).

**Food group**	**1st 3FQ**	**2nd 3FQ**	**Rs^a^**	**(ICC)^b^**
	**g/day**	**g/day**		
Plant-based meat or dairy alternatives	0 (0, 4)^c^	0 (0, 5.35)^c^	0.757^**^	0.788^**^
Fermented cheese	10.2 (1.95, 26.4)	15.77 (4.2, 34)	0.629^**^	0.708^**^
Fermented yogurt and milk	47.5 (10, 133.3)	63 (10, 136.25)	0.731^**^	0.768^**^
Fermented dairy products (animal-based)	74.86 (24.58, 174.35)	88.00 (29.8, 186.9)	0.717^**^	0.773^**^
Fermented pulses and legumes	0.89 (0.25, 3.10)	0.84 (0.25, 2.96)	0.624^**^	0.774^**^
Fermented meat or fish	2.15 (0.8, 5.46)	1.50 (0.8, 5.46)	0.637^**^	0.756^**^
Fermented vegetables	5.84 (2, 18.24)	7.46 (2, 18.24)	0.682^**^	0.736^**^
White or white sourdough bread	25.42 (4.13, 79.43)	32.74 (4.62, 93.88)	0.653^**^	0.728^**^
Whole grain or whole grain sourdough bread	11.88 (1.95, 50.84)	11.48 (1.59, 52.89)	0.728^**^	0.720^**^
Bread	55.16 (20.65, 132.28)	67.52 (25.84, 148.14)	0.625^**^	0.720^**^
Fermented cereals (trahana and all breads)	57.25 (21.35, 135.12)	68.88 (26.22, 153.18)	0.626^**^	0.717^**^
Chocolate	19.82 (4.72, 42.01)	19.82 (5.66, 40.59)	0.678^**^	0.737^**^
Fermented non-alcoholic soft beverages	3.56 (1.78, 10.68)	4.45 (1.78, 12.46)	0.780^**^	0.863^**^
Vinegar-all types	2.07 (0.49, 6.95)	3.10 (0.74, 9.45)	0.710^**^	0.837^**^
Coffee	60 (19.2, 159.48)	62.58 (25.14, 169.83)	0.579^**^	0.777^**^
Tea	4.74 (0, 29.63)	4.74 (0, 48.59)	0.773^**^	0.876^**^
Cocoa beverages	9.48 (4.74, 23.7)	9.48 (4.74, 16.59)	0.610^**^	0.603^**^
Beer or cider	35 (16.5, 105)	41.25 (16.5, 115)	0.802^**^	0.894^**^
Wine-all types	15 (6.25, 50)	22.5 (10, 62.5)	0.781^**^	0.882^**^
Strong spirits	5.92 (2.37, 17.75)	5.92 (2.37, 8.88)	0.714^**^	0.759^**^

^**^Correlation is significant at the 0.01 level (two-tailed).

3FQ, fermented food frequency questionnaire.

^a^Spearman's correlation coefficient was used to determine the level of correlation between the two repetitions of the 3FQ.

^b^Additionally, intraclass correlation coefficient (ICC) was used to determine intra-class correlation between them. Spearman's correlation coefficient is interpreted as follows 0.1–0.3: weak correlation, 0.3–0.5: medium correlation and 0.5–1.0 strong correlation. ICC is interpreted, respectively: ICC <0.5 poor, 0.5 ≤ ICC <0.75 moderate, 0.75 ≤ ICC <0.9 good and ICC ≥ 0.9 excellent reliability; Intakes are presented as g/day and each main food group presented in this table sums the answers of the subgroups answered in the 3FQ. For example, food group “Fermented cheese” sums the consumptions that refer to the respective categories: “Hard Cheese (i.e., Parmigiano Reggiano, Grana Padano, Gruyere, Manchego, Cheddar, Pecorino),” “Semi-hard cheese (i.e., Edam, Gouda),” “Semi-hard cheese (i.e., Gorgonzola, Blue cheese),” “Soft and/or Fresh cheese (i.e., Feta, Quark)” and “Soft and/or Fresh cheese (i.e., Chevre, Camembert, Brie).”

^c^Variables are presented as median (25th−75th percentile) following their skewed distribution.

**Table 4:** Spearman's correlation and ICC coefficients to assess repeatability of the 3FQ^a^ on the whole sample.

**Food group**	**1st 3FQ** ^**b**^		**2nd 3FQ** ^**b**^		**Rs^c^**
	**(** ***n*** **)**	**Frequency/day**	**(** ***n*** **)**	**Frequency/day**	
Plant-based meat or dairy alternatives^d^	12,622	0 (0, 0.05)^d^	1,248	0 (0, 0.06)	0.763^**^
Fermented cheese	12,605	0.51 (0.16, 1)	1,248	0.67 (0.21, 1.13)	0.647^**^
Fermented yogurt and milk	12,626	0.26 (0.07, 0.85)	1,247	0.42 (0.07, 0.85)	0.731^**^
Fermented dairy products (animal-based)	12,635	1.01 (0.42, 1.75)	1,248	1.21 (0.61, 1.98)	0.675^**^
Fermented pulses and legumes	8,392	0.05 (0.02, 0.21)	825	0.05 (0.02, 0.15)	0.678^**^
Fermented meat or fish	11,225	0.12 (0.05, 0.25)	1,135	0.09 (0.05, 0.23)	0.630^**^
Fermented vegetables	11,528	0.23 (0.09, 0.63)	1,177	0.23 (0.09, 0.63)	1.000^**^
White or white sourdough bread	11,496	0.42 (0.07, 1)	1,169	0.42 (0.07, 1)	0.705^**^
Whole grain or whole grain sourdough bread	11,627	0.21 (0.04, 0.64)	1,181	0.21 (0.04, 0.66)	0.768^**^
Bread	11,545	0.85 (0.36, 1.21)	1,174	0.89 (0.44, 1.11)	0.639^**^
Fermented cereals (trahana and all breads)	11,598	0.87 (0.42, 1.21)	1,155	0.92 (0.46, 1.21)	0.648^**^
Chocolate	3,541	0.23 (0.09, 0.64)	327	0.25 (0.1, 0.66)	0.697^**^
Fermented non-alcoholic soft beverages	8,421	0.04 (0.02, 0.07)	899	0.04 (0.02, 0.07)	0.710^**^
Vinegar-all types	10,064	0.23 (0.08, 0.66)	999	0.28 (0.1, 0.73)	0.728^**^
Coffee	4,881	1 (0.64, 1.06)	1,025	1 (0.68, 1.07)	0.639^**^
Tea	5,678	0.02 (0, 0.17)	587	0.02 (0, 0.15)	0.743^**^
Cocoa beverages	7,641	0.05 (0.02, 0.1)	775	0.05 (0.02, 0.07)	0.602^**^
Beer or cider	8,100	0.09 (0.05, 0.23)	824	0.09 (0.05, 0.23)	0.805^**^
Wine-all types	6,083	0.09 (0.05, 0.21)	831	0.12 (0.06, 0.28)	0.773^**^
Strong spirits	6,083	0.02 (0.02, 0.05)	604	0.02 (0.02, 0.05)	0.686^**^

Results are expressed as frequency/day.

Significance at alpha 5% level (two-tailed), ^**^Correlation is significant at the 0.01 level (two-tailed).

^a^3FQ, fermented food frequency questionnaire.

^b^n, study sample population.

^c^Spearman's correlation coefficient was used to determine the level of correlation between the two repetitions of the 3FQ. Intakes are presented as frequency/day and each main food group presented in this table sums the answers of the particular subgroups answered in the 3FQ. For example, food group “Fermented cheese” sums the consumptions that refer to the respective categories: “Hard Cheese (i.e., Parmigiano Reggiano, Grana Padano, Gruyere, Manchego, Cheddar, Pecorino),” “Semi-hard cheese (i.e., Edam, Gouda),” “Semi-hard cheese (i.e., Gorgonzola, Blue cheese),” “Soft and/or Fresh cheese (i.e., Feta, Quark)” and “Soft and/or Fresh cheese (i.e., Chevre, Camember, Brie).”

^d^Variables are presented as median (25th−75th percentile) following their skewed distribution.

**Figure 1:**
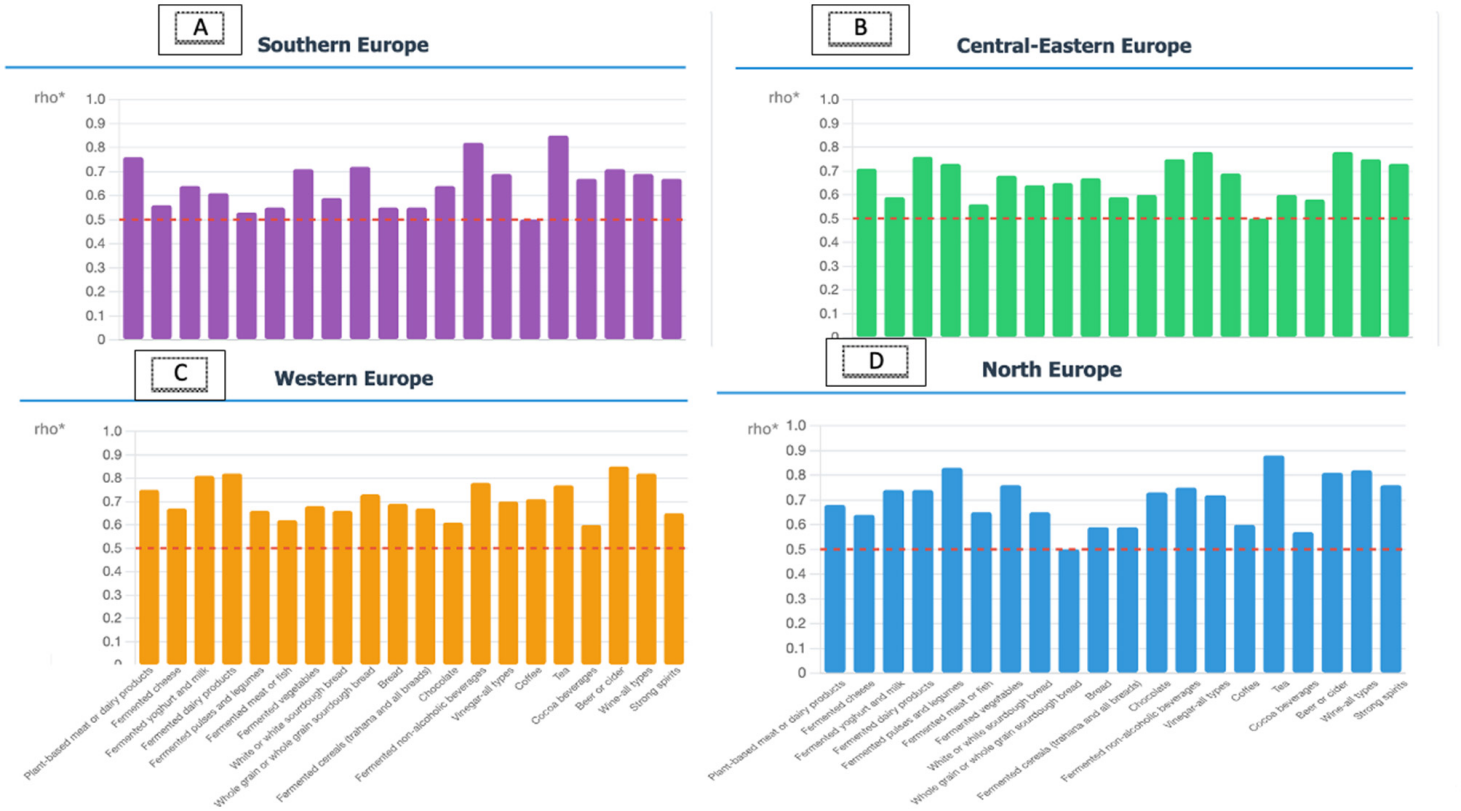
Spearman's correlation coefficients to check repeatability of the 3FQ across European Regions. **(A)** Southern Europe, **(B)** Central-Eastern Europe, **(C)** Western Europe, and **(D)** North Europe; Data collected using: 372 (29.81%) Central-Eastern Europe; 338 (27.08%) Western Europe; 424 (33.97%) Southern Europe and 114 (9.13%) from Northern Europe. *Spearman's correlation coefficient was used to determine the level of correlation between the 2 repetitions of the 3FQ.

All the aforementioned tests were conducted to assess the overall reliability of the 3FQ for the entire sample across the four European regions. To assess the 3FQ per European region, all tests were repeated separately for each region, and Spearman's correlation coefficient results are presented in Figure 1, where a strong test-retest reliability was found across all four regions, ranging from 0.6 to 0.8 for most fermented foods. Results were based on 424 participants (34%) from Southern Europe followed by 372 (29.8%) from Central-Eastern Europe, 338 (27.1%) from Western Europe, and 114 participants (9.13%) from Northern Europe. Northern Europe exhibited the most variable pattern with coefficients ranging from ~0.5 to 0.9, though this variability may be influenced by the smaller sample size in this region.

Details regarding consumption between the two responses (in grams per day and total consumers), Spearman correlation and ICC can be found in Supplementary Tables 2–9. Overall, the ICC test resulted in moderate to excellent reliability for the consumption estimates, expressed as grams per day, for most food groups in the European regions. Several exceptions were found for the food groups: “tea” (average ICC = 0.305, NS), “fermented meat and fish” (average ICC = 0.455, *P* < 0.05), “whole grain or whole grain sourdough bread” (average ICC = 0.472, *P* < 0.01) in the Northern European region, for “fermented non-alcoholic beverages” in Central-Eastern European region (average ICC = 0.329, NS), and for “Cocoa beverages” in Southern European region (average ICC = 0.492, *P* < 0.01).

**Figure 2:**
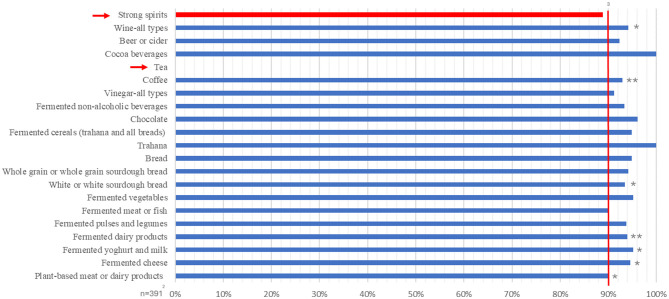
%Within the agreement interval to assess validity of the 3FQ^1^ on the whole Sample using 24 h. *Correlation is significant at the 0.05 level (two-tailed), **Correlation is significant at the 0.001 level (two-tailed). ^1^3FQ: fermented food frequency questionnaire. ^2^n = 391 (validity study sample population). ^3^% within the agreement interval between the results of the 3FQ and the 24 h per food group. Bland and Altman analysis was used to determine the percentage of agreement between the two methods. Red bars indicate the food subcategories that an agreement was not obtained through Bland-Altman analysis.

Figure 2 outlines the repeatability results comparing the 3FQ questionnaire with the 24 h, which was used as the gold standard to assess the actual consumption of the examined fermented food items and groups. The detailed results are given in Supplementary Table 10 and Supplementary Figures 1–4. Significant associations were found for simple Spearman's correlation analysis across the food groups in the association testing but relatively few reached statistical significance. However, the results of the Bland-Altman analysis show that most food groups exhibit excellent agreement between the 3FQ and 24 h, with most categories having more than 90% of values falling within the agreement interval. This result indicates good general validity. Actually, for the food groups “fermented yogurt and milk,” “fermented vegetables,” “fermented cereals [trahana and all breads (and types)],” “chocolate” and “cocoa beverages” and the individual food items “hard cheese (i.e., Parmigiano Reggiano),” “semi-hard cheese (i.e., edam),” “semi-hard cheese (i.e., gorgonzola),” “soft and/or fresh cheese (i.e., feta),” “fermented yogurt,” “olives,” “fermented cabbage (i.e., sauerkraut),” all types of chocolate, apple, grape and balsamic vinegars, “espresso coffee,” “Arabic coffee,” and “instant coffee,” for which the % agreement outreached 95%. The only exceptions were the food groups “other fermented vegetables” and “strong spirits,” which reached a marginally lower level of agreement, and “tea,” which could not be tested due to a lack of sufficient number of responses for conducting the test. Similar findings were observed for the food group analysis by European region with all regions having insufficient responses to conduct tests for “strong spirits” and “tea.” However, some foods were validated only for certain regions, such as “fermented pulses and legumes” (validated only for Northern Europe), “Vinegar-all-types” (validated for Southern Europe and Central-Eastern Europe), and “fermented non-alcoholic beverages” (validated only for Western Europe). Conversely, other foods were not validated in specific regions, such as “fermented meat and fish” (not validated in Southern Europe) and “wine-all-types” (not validated for Central-Eastern Europe).

## Discussion

Fermented foods are widely consumed worldwide, but to our knowledge, there is no validated tool specifically designed to investigate consumption of this complex but important food type. The current study investigated the validity of the 3FQ, a FFQ developed specifically to assess fermented food consumption in European countries. Overall, study results showed that the 3FQ reliably captured consumption of fermented foods, yielding consistent results for repeated estimates in terms of quantity and frequency of intake across all fermented food groups. The agreement of the 3FQ compared to repeated 24 h showed that the validity of the 3FQ was generally good, although for some food groups and items with infrequent consumption, such as fermented fish, the validity was limited.

Among the fermented foods investigated, dairy products, coffee, and bread were the best validated categories, showing good agreement between 3FQ and 24 h and significant correlations between the dietary assessment methods. These foods were consumed regularly by the majority of the sample population and thus provided the most robust validation data. Food groups that were less widely consumed tended to show lower correlation and agreement between the methods, such as fermented vegetables and fermented fish, potentially due to high intraindividual variability, as reported by other researchers for the same non-fermented food groups (30). Some infrequently consumed foods, such as plant-based meat and dairy alternatives (*n* = 10 consumers), showed excellent validity; however, the reliability of these results is very limited and cannot be generalized most probably due to low consumption as the effect of consumption frequency on the performance of FFQs for estimating fermented food intake has been reported previously (8), with robust results for regularly consumed food items (coffee, bread, and fermented dairy) but poor results for infrequently consumed items (buttermilk and quark).

Some differences in consumption patterns between the four European regions sampled were observed in terms of the numbers of consumers and non-consumers, which limited the universal validation of certain foods and food groups across the regions (for example, “fermented pulses and legumes” were only validated in Northern Europe). However, it was remarkable that consistent validity results were obtained for all four European regions, when assessed separately, for the most fermented foods and beverages. This observation may reflect the observation that while there are regional differences in specific fermented foods consumed, consumption of fermented food groups is more consistent (31). This was one of the motivations for providing regional examples of foods within the food groups in the 3FQ. The repeatability for the 3FQ was broadly comparable across the four regions, although some exceptions for less frequently consumed foods in Northern Europe may be an artifact of the smaller sample size for this region.

As recommended by Suba et al. (32), this epidemiological survey collected dietary data from both short-term (24 h) and long-term (FFQs) instruments on the entire study population to maximize the tool strength, which for this tool was to map fermented food intakes, in terms of frequency and quantity, in the European Region. It also draws on the observation that long-term dietary exposure assessment is best performed using food frequency data, particularly for foods consumed intermittently (33). For the validation of the 3FQ, and in the absence of valid recovery biomarkers for fermented foods, 24 h with the AMPM with specific food pictures for each country, were used, as these are considered the best estimates of absolute dietary intakes (34). Food diaries, although considered superior in portion accuracy, do not accurately depict usual intake, as individuals often alter their choices when they must report them, leading to information bias (11). Daily median intakes from the 3FQ for many food groups were consistently lower compared to those reported in the 24 h, as the frequency of consumption was accounted for, thereby adjusting portion size. The use of consumption frequency is valuable for estimating long-term intake, as it helps decrease the probability of overestimating intakes by accounting for intra-individual variability between days. The strength of the specific 3FQ tool lies in its explicit consideration of both the frequency of consumption and portion size of specific foods not regularly consumed, utilizing a wide range of validated food pictures for each food group included. Portion size misinterpretation has been reported as the main error contributing to intake misestimations of specific foods and beverages, with the second being the omission of foods, which is particularly prevalent for vegetables and foods not regularly consumed (35). In contrast, while the 24 h is used as the reference assessment method for the validity, as it captures detailed information on a single day (or mean of 2 days), it may overestimate certain foods if the recall captures foods only eaten occasionally.

The 3FQ was developed as an adaptable tool that aims to capture the specificities of the different fermented foods and beverages consumed across countries with diverse dietary behaviors in a single, common approach. The questionnaire was tailored for all study populations and was piloted to test its comprehensibility and applicability. One challenge was to ensure responders' understanding of what can be considered a fermented food. This limitation was addressed by providing country-specific examples for each main food category and by pilot testing the study prior to its launch. Similarly, the portion sizes and frequency of intake categories were adjusted to encompass the variety of dietary habits expected. The Bland-Altman results confirm that the selected categories were generally appropriate for the typical consumption patterns observed in the validation study. There were some exceptions, but these tended to be for subgroups that showed infrequent consumption. A strength of the validation study was that it was conducted across all European regions within a single, limited timeframe, thereby minimizing dietary variation associated with seasonality and time-specific contextual factors. However, results from Northern Europe should be interpreted with caution, since although an appropriate sample was obtained for Northern Europe, the smaller sample size compared to the other regions may limit the generalizability of these findings. Overall, although representative samples by age group and sex were acquired across all European Regions, total sample sizes greatly differed and will therefore be accounted for by taking weighted averages, as previously reported (18) prior to presenting final fermented food intakes. Also, while cultural differences across countries remain, simultaneous implementation contributes to the overall comparability of the results. The stability of the validation results across seasons has not yet been ascertained but could be the focus of a follow-up study.

The recruitment strategy for the current study aimed to have a very wide and diverse selection of participants; however, the validation sub-population showed a relatively high proportion of educated participants with a relevant scientific background and uneven participation across the selected European countries. This is a limitation that is difficult to overcome in this validation and is a consequence of convenience sampling methodology. While this is standard practice in tool validation studies, we cannot rule out that highly educated participants may be overrepresented. Also, the participants with nutrition backgrounds represented ~38% of the total validation sample, and although their expertise may have decreased recall bias, this characteristic may limit the external validity of our findings. In the total sample participants with nutrition backgrounds represent 30% of the total population and will be separately addressed through sensitivity analyses when whole sample intakes will be derived, as written in the protocol (18) for complete transparency. Substantial sample size and the use of various means for sampling (social media and direct contact with the community) were used to mitigate bias. Remarkably, the results of the regional analysis demonstrated the robustness of the questionnaire's repeatability and validity for regularly consumed food groups across all European regions, regardless of sample size. Conversely, a limitation in the study was the varying sample sizes across the less frequently consumed food items, which affected the reliability and validity of the questionnaire. Food consumed by <10% of participants may lead to an artificially inflated correlation, due to the predominance of zero values (36). Although this was mitigated by the robust validation steps taken through specific power sample size computations (18), results of sporadically consumed fermented foods and/or traditional regional foods should still be interpreted with caution. Also, population demographic differences were found within the repeatability responders, such as educational attainment and background, which may limit the generalizability of results. The wide range of correlation strengths suggests that 3FQ performs better for some food categories than others. The use of additional 24 h repeats could have potentially improved the assessment of some less frequently consumed fermented foods and beverages (10). It is important to note that, based on the aim of the 3FQ, which is to assess type, frequency and quantity (estimation) of fermented foods by European Region, and it's assessed validity, information collected can be used by each European Region to further capture emerging, non-traditional fermented foods entering their markets from other regions.

With a growing market for new fermented products in Europe including from other cultures, it may be necessary to update and adapt the 3FQ in the future. Indeed, while the 3FQ addressed the dietary assessment of commonly consumed traditional fermented foods and beverages, the tool does not exhaustively list all fermented products across each food category. Nevertheless, the tool remains appropriate for capturing the most frequently consumed fermented foods across the main food groups. Compared to existing FFQs that may capture the intake of certain commonly consumed fermented foods (e.g., bread, cheese, wine) as part of the whole dietary intake, the 3FQ differentiates certain characteristics of fermented foods (for example method of dough fermentation) that could lead to misclassification of foods as “fermented” in a general FFQ. The validity of the 3FQ for assessing the intake of fermented foods, which are a prevalent item in European diets, is required to enable its use in evaluating the health impact of fermented foods. as with any validated tool however, the 3FQ has been validated in total and not in subjections. despite the high correlations and agreements within specific fermented food groups, if parts of the tool are used, it will require validation.

## Conclusion

The 3FQ is a robust tool for estimating the usual consumption of fermented food groups in different European regions and is the first validated tool that can assess intake of all main fermented food categories. The questionnaire provided reliable and accurate estimates of the consumption frequency for most fermented foods. The use of the tool for assessing the intake of less frequently consumed fermented foods should be limited or used in conjunction with biomarkers for fermented foods when these are available.

## Data Availability

The raw data supporting the conclusions of this article will be made available by the authors, without undue reservation.

## References

[1] Tamang JP Cotter PD Endo A Han NS Kort R Liu SQ. Fermented foods in a global age: east meets West. Compr Rev Food Sci Food Saf. (2020) 19:184–217. 10.1111/1541-4337.12520 33319517

[2] Lee C-H Ahn J Son H-S. Ethnic fermented foods of the world: an overview. J Ethnic Foods. (2024) 11:39. 10.1186/s42779-024-00254-2

[3] Praagman J Dalmeijer GW van der Schouw YT Soedamah-Muthu SS Verschuren WMM Bueno-de-Mesquita HB. The relationship between fermented food intake and mortality risk in the European prospective investigation into cancer and nutrition-Netherlands cohort. Br J Nutr. (2015) 113:498–506. 10.1017/S0007114514003766 25599866

[4] SaeidiFard N Djafarian K Shab-Bidar S. Fermented foods and inflammation: a systematic review and meta-analysis of randomized controlled trials. Clin Nutr ESPEN. (2020) 35:30–9. 10.1016/j.clnesp.2019.10.010 31987119

[5] Zhang XF Qi Y Zhang YP Deng JL Chen XL Li RN. Fermented foods and metabolic outcomes in diabetes and prediabetes: a systematic review and meta-analysis of randomized controlled trials. Crit Rev Food Sci Nutr. (2024) 64:9514–31. 10.1080/10408398.2023.2213770 37204758

[6] Naska A Lagiou A Lagiou P. Dietary assessment methods in epidemiological research: current state of the art and future prospects. F1000Res. (2017) 6:926. 10.12688/f1000research.10703.1 28690835 PMC5482335

[7] Fujihashi H Sasaki S. Identification and estimation of the intake of fermented foods and their contribution to energy and nutrients among Japanese adults. Public Health Nutr. (2024) 27:e153. 10.1017/S1368980024000405 38361454 PMC11617413

[8] Li KJ Brouwer-Brolsma EM Burton KJ Vergères G Feskens EJM. Prevalence of fermented foods in the Dutch adult diet and validation of a food frequency questionnaire for estimating their intake in the NQplus cohort. BMC Nutr. (2020) 6:69. 10.1186/s40795-020-00394-z 33292738 PMC7712622

[9] Pertziger E von Ah U Bochud M Chatelan A Haldemann J Hillesheim E. Classification and estimation of dietary live microorganisms and fermented foods intake in Swiss adults. J Nutr. (2025). 10.1016/j.tjnut.2025.05.042 40451611

[10] Shim J-S Oh K Kim HC. Dietary assessment methods in epidemiologic studies. Epidemiol Health. (2014) 36:e2014009. 10.4178/epih/e2014009 25078382 PMC4154347

[11] Lam YY Ravussin E. Analysis of energy metabolism in humans: a review of methodologies. Mol Metab. (2016) 5:1057–71. 10.1016/j.molmet.2016.09.005 27818932 PMC5081410

[12] Millen AE Midthune D Thompson FE Kipnis V Subar AF. The National Cancer Institute diet history questionnaire: validation of pyramid food servings. Am J Epidemiol. (2006) 163:279–88. 10.1093/aje/kwj031 16339051

[13] Subar AF Dodd KW Guenther PM Kipnis V Midthune D McDowell M. The food propensity questionnaire: concept, development, and validation for use as a covariate in a model to estimate usual food intake. J Am Diet Assoc. (2006) 106:1556–63. 10.1016/j.jada.2006.07.002 17000188

[14] Berchtold A. Test–retest: agreement or reliability? Methodol Innov. (2016) 9:2059799116672875. 10.1177/2059799116672875

[15] Cade J Thompson R Burley V Warm D. Development, validation and utilisation of food-frequency questionnaires - a review. Public Health Nutr. (2002) 5:567–87. 10.1079/PHN2001318 12186666

[16] Li KJ Burton-Pimentel KJ Brouwer-Brolsma EM Blaser C Badertscher R Pimentel G. Identifying plasma and urinary biomarkers of fermented food intake and their associations with cardiometabolic health in a Dutch observational cohort. J Agric Food Chem. (2023) 71:4426–39. 10.1021/acs.jafc.2c05669 36853956 PMC10021015

[17] Li KJ Brouwer-Brolsma EM Burton-Pimentel KJ Vergères G Feskens EJM. A systematic review to identify biomarkers of intake for fermented food products. Genes Nutr. (2021) 16:5. 10.1186/s12263-021-00686-4 33882831 PMC8058972

[18] Magriplis E Kotopoulou S Adamberg S Burton-Pimentel KJ Kitryte-Syrpa V Laranjo M. Protocol for the development and validation of an online fermented foods frequency questionnaire (3FQ) for the assessment of fermented foods consumption patterns across European regions. JMIR Res Protoc. (2025) 14:e69212. 10.2196/69212 40920447 PMC12455144

[19] Action CA20128. Cost. Available online at: https://www.cost.eu/actions/CA20128 (Accessed May 28, 2025).

[20] Browse by EuroVoc - EUR-Lex. Available online at: https://eur-lex.europa.eu/browse/eurovoc.html?params=72%2C7226 (Accessed July 14, 2025).

[21] Lee KM Lee J Chung CY Ahn S Sung KH Kim TW. Pitfalls and important issues in testing reliability using intraclass correlation coefficients in orthopaedic research. Clin Orthop Surg. (2012) 4:149–55. 10.4055/cios.2012.4.2.149 22662301 PMC3360188

[22] Willett W. Nutritional Epidemiology. 3rd ed. (2012; online edn). Oxford Academic (2013). 10.1093/acprof:oso/9780199754038.001.0001

[23] Mishra P Pandey CM Singh U Gupta A Sahu C Keshri A. Descriptive statistics and normality tests for statistical data. Ann Card Anaesth. (2019) 22:67–72. 10.4103/aca.ACA_157_18 30648682 PMC6350423

[24] Bewick V Cheek L Ball J. Statistics review 7: correlation and regression. Crit Care. (2003) 7:451. 10.1186/cc2401 14624685 PMC374386

[25] Schober P Boer C Schwarte LA. Correlation coefficients: appropriate use and interpretation. Anesth Analg. (2018) 126:1763–8. 10.1213/ANE.0000000000002864 29481436

[26] Liljequist D Elfving B Roaldsen KS. Intraclass correlation – a discussion and demonstration of basic features. PLoS ONE. (2019) 14:e0219854. 10.1371/journal.pone.0219854 31329615 PMC6645485

[27] McHugh ML. Interrater reliability: the kappa statistic. Biochem Med. (2012) 22:276–82. 10.11613/BM.2012.031 PMC3900052 23092060

[28] Bland JM Altman DG. Statistical methods for assessing agreement between two methods of clinical measurement. Lancet. (1986) 1:307–10. 10.1016/S0140-6736(86)90837-8 2868172

[29] Fallaize R Forster H Macready AL Walsh MC Mathers JC Brennan L. Online dietary intake estimation: reproducibility and validity of the food4me food frequency questionnaire against a 4-day weighed food record. J Med Internet Res. (2014) 16:e3355. 10.2196/jmir.3355 25113936 PMC4147714

[30] Souverein OW Dekkers AL Geelen A Haubrock J de Vries JH Ocké MC. Comparing four methods to estimate usual intake distributions. Eur J Clin Nutr. (2011) 65:S92–101. 10.1038/ejcn.2011.93 21731012

[31] Tamang JP Watanabe K Holzapfel WH. Review: diversity of microorganisms in global fermented foods and beverages. Front Microbiol. (2016) 7:377. 10.3389/fmicb.2016.00377 27047484 PMC4805592

[32] Subar AF Freedman LS Tooze JA Kirkpatrick SI Boushey C Neuhouser ML. Addressing current criticism regarding the value of self-report dietary data. J Nutr. (2015) 145:2639–45. 10.3945/jn.115.219634 26468491 PMC4656907

[33] Willett W. Recall of remote diet. In: Nutritional Epidemiology. 3rd, ed. (2012; online edn). Oxford Academic (2013). 10.1093/acprof:oso/9780199754038.003.0007

[34] Park Y Dodd KW Kipnis V Thompson FE Potischman N Schoeller DA. Comparison of self-reported dietary intakes from the automated self-administered 24-h recall, 4-d food records, and food-frequency questionnaires against recovery biomarkers. Am J Clin Nutr. (2018) 107:80–93. 10.1093/ajcn/nqx002 29381789 PMC5972568

[35] Whitton C Ramos-García C Kirkpatrick SI Healy JD Dhaliwal SS Boushey CJ. Systematic review examining contributors to misestimation of food and beverage intake based on short-term self-report dietary assessment instruments administered to adults. Adv Nutr. (2022) 13:2620–65. 10.1093/advances/nmac085 36041186 PMC9776649

[36] Zhang S Midthune D Guenther PM Krebs-Smith SM Kipnis V Dodd KW. A new multivariate measurement error model with zero-inflated dietary data, and its application to dietary assessment. Ann Appl Stat. (2011) 5:1456–87. 10.1214/10-AOAS446 21804910 PMC3145332

