# Supplementary material for: Validation of the fermented food frequency questionnaire to assess consumption across four European regions: a study within the promoting innovation of fermented foods cost action
Source: Front Nutr. 2025 Oct 29;12:1667653. doi: 10.3389/fnut.2025.1667653 (PMC12608085; doi:10.3389/fnut.2025.1667653)
Supplement: Supplementary file 1 [file Supplementary_file_1.docx]

Supplementary Table S 1. Socio-demographic & anthropometric characteristics in the study population by repetition of the 3FQ^1^

|  | 1st 3FQ (n=12 646)^2^ | 2nd 3FQ (n=1, 248)^2^ | p-value^3^ |
| --- | --- | --- | --- |
| Sex (females), N (%) | 8, 588 (67.92%) | 856 (68.59%) | <0.001^4,5^ |
| Age, mean (sd) | 41.1 (14.7) | 42.42 (13.95) | 0.945 |
| BMI^6^ | 24.5 (5.3) | 24.43 (4.85) | 0.747 |
| Marital status, N (%) | 12, 566 (100%) | 1, 243 (100%) | <0.001 |
| Married/ living with a spouse | 7, 085 (56.38%) | 747 (60.10%) |  |
| Single | 4, 118 (32.77%) | 398 (32.02%) |  |
| Divorced/separated/widowed | 819 (6.52%) | 76 (6.11%) |  |
| Prefer not to answer, N (%) | 544 (4.33%) | 22 (1.77%) |  |
| Educational level, N (%) | 12, 584 (100%) | 1, 245 (100%) | <0.001 |
| Doctorate (PhD) | 1, 996 (15.86%) | 271 (21.77%) |  |
| Masters (MSc) | 2, 780 (22.09%) | 329 (26.43%) |  |
| University diploma | 4, 145 (32.94%) | 371 (29.80%) |  |
| 9-12 years of study | 2, 899 (23.04%) | 198 (15.90%) |  |
| 6-9 years of study | 325 (2.58%) | 29 (2.33%) |  |
| <6 years of study (primary education, elementary school) | 244 (1.94%) | 42 (3.37%) |  |
| Prefer not to answer, N (%) | 195 (1.55%) | 5 (0.40%) |  |
| Relevant scientific background (Yes), N (%) | 3, 873 (30.74%) | 476 (38.17%) | <0.001 |
| Employment status (employed), N (%) | 9, 099 (73.93%) | 949 (77.03%) | <0.001 |
| Smoking status, N (%) | 12, 567 (100%) | 1, 242 (100%) | <0.001 |
| Non-smoker, | 8, 695 (69.19%) | 884 (71.18%) |  |
| Smoker | 2, 584 (20.56%) | 201 (16.18%) |  |
| Ex-smoker | 1, 288 (10.25%) | 157 (12.64%) |  |
| Food allergies-intolerances (Yes), N (%) | 1983 (15.68%) | 184 (14.74%) | <0.001 |
| Last answered question, mean (sd) | 78.96 (1.01) | 78.96 (1.01) | 0.436 |

Significance at alpha 5% level; ^1^3FQ, Fermented Food Frequency Questionnaire, ^2^ n: study sample population. ^3^p-values indicate whether there are statistically significant differences between variables that are present in the table, by 3FQ repetition.^4^ Age, BMI and Last answered question variables were examined using the t-test method for two samples and for the rest of the variables (categorical ones), chi-square test was used. ^5^Significance level set at alpha 5%. ^6^BMI: Body Mass Index, calculated as weight (kg) divided by height squared (cm2).

Supplementary Table S2: Spearman’s correlation and ICC coefficients to check Repeatability of the 3FQ^1^ for Northern Europe

|  | **1st 3FQ^1^ n=2315)^2^** | | **2nd 3FQ (n=114)^2^** | | **r** | **Rs^3^** | **(ICC)^3^** |
| --- | --- | --- | --- | --- | --- | --- | --- |
|  | (n) | gr/day | (n) | gr/day |  |  |  |
| **Plant-based meat or dairy alternatives ^4^** | 2, 305 | 0 (0, 4)^5^ | 114 | 0 (0, 2.14) | 115 | 0.679** | 0.920** |
| **Fermented cheese** | 2, 285 | 10.2 (1.6, 28.14) | 114 | 18.71 (4.6, 38.17) | 115 | 0.649** | 0.710** |
| **Fermented yoghurt and milk** | 2, 313 | 55.65 (10.6, 160) | 114 | 79.3 (21, 224) | 115 | 0.738** | 0.805** |
| **Fermented dairy products** | 2, 313 | 79.06 (24.09, 200.4) | 114 | 124.26 (41, 271.79) | 115 | 0.742** | 0.827** |
| **Fermented pulses and legumes** | 1, 497 | 1.18 (0.44, 3.35) | 70 | 1.15 (0.49, 2.46) | 61 | 0.830** | 0.742** |
| **Fermented meat or fish** | 1, 831 | 1.3 (0.74, 4.26) | 93 | 1.05 (0.52, 3.02) | 87 | 0.652** | 0.455* |
| **Fermented vegetables** | 1, 966 | 4.94 (1.56, 18.00) | 104 | 7.06 (1.79, 21.55) | 103 | 0.760** | 0.681** |
| **White or white sourdough bread** | 2, 073 | 30.08 (6.85, 79.43) | 109 | 49.05 (11.32, 123.37) | 103 | 0.650** | 0.740** |
| **Whole grain or whole grain sourdough bread** | 2, 055 | 26.51 (6.39, 79.74) | 109 | 37.19 (10.58, 98.30) | 102 | 0.508** | 0.472** |
| **Bread** | 2, 078 | 75.86 (26.12, 168.16) | 109 | 99.03 (38.16, 217.27) | 103 | 0.596** | 0.651** |
| **Fermented cereals (trahana and all breads)** | 2, 089 | 77.05 (27.32, 169.29) | 109 | 99.03 (41, 217.97) | 103 | 0.596** | 0.651** |
| **Chocolate** | 2, 147 | 10.86 (4.72, 30.21) | 109 | 10.62 (4.72, 30.21) | 107 | 0.729** | 0.892** |
| **Fermented non-alcoholic soft beverages** | 1, 127 | 4.75 (2.97, 12.46) | 59 | 5.79 (3.56, 13.80) | 50 | 0.746** | 0.832** |
| **Vinegar-all types** | 1, 556 | 0.74 (0.30, 3.10) | 87 | 1.0091 (0.30, 2.81) | 82 | 0.723** | 0.885** |
| **Coffee** | 1, 920 | 73.05 (12.6, 241.74) | 97 | 137.2125 (30.6, 382.2) | 96 | 0.599** | 0.793** |
| **Tea** | 768 | 9.48 (1.58, 39.30) | 30 | 9.48 (4.74, 35.55) | 23 | 0.882** | **0.305** |
| **Cocoa beverages** | 1, 090 | 9.48 (4.74, 16.59) | 53 | 9.48 (5.93, 16.59) | 45 | 0.578** | 0.637** |
| **Beer or cider** | 1, 509 | 45 (16.5, 115.5) | 71 | 48.1 (16.5, 140) | 67 | 0.807** | 0.941** |
| **Wine-all types** | 1, 545 | 18.75 (7.5, 52.50) | 84 | 27.5 (11.25, 58.75) | 81 | 0.822** | 0.842** |
| **Strong spirits** | 1, 152 | 5.92 (2.37, 17.75) | 60 | 3.55 (2.37, 17.75) | 57 | 0.762** | 0.552* |

* p< 0.05, **p< 0.01; ^1^ 3FQ, Fermented Food Frequency Questionnaire; ^2^ n: study sample population.;^3^ Spearman's correlation coefficient was used to determine the level of correlation between the 2 repetitions of the 3FQ. ^3^Additionally, Intraclass Correlation Coefficient (ICC) was used to determine intra-class correlation between them. Spearman's correlation coefficient is interpreted as follows 0.1 - 0.3: weak correlation, 0.3-0.5: medium correlation and 0.5-1.0 strong correlation. ICC is interpreted respectively: ICC<0.5 poor, 0.5≤ICC<0.75 moderate, 0.75≤ICC<0.9 good and ICC≥0.9 excellent reliability.; ^4^ Intakes are presented as g/day and each main food group presented in this table sums the answers of the particular subgroups answered in the 3FQ. For example, food group “Fermented cheese” sums the consumptions that refer to the respective categories: “Hard Cheese (i.e. Parmigiano Reggiano, Grana Padano, Gruyere, Manchego, Cheddar, Pecorino)”, “Semi-hard cheese (i.e. Edam, Gouda)”, “Semi-hard cheese (i.e. Gorgonzola, Blue cheese)”, “Soft and/or Fresh cheese (i.e. Feta, Quark)” and “Soft and/or Fresh cheese (i.e. Chevre, Camembert, Brie)”; ^5^ Variables are presented as median (25th–75th percentile) following their skewed distribution. Repeatability was checked for 20 food groups in total since bread types and dairy types were also separately assessed (20 instead of 16).

Supplementary Table S3: Spearman’s correlation to check Repeatability of the 3FQ^1^ for Northern Europe for frequency/day

|  | **1st 3FQ (n=2315)^2^** | | **2nd 3FQ (n=114)^2^** | | **r** | **Rs^3^** |
| --- | --- | --- | --- | --- | --- | --- |
|  | (n) | frequency/day | (n) | frequency/day |  |  |
| **Plant-based meat or dairy alternatives ^4^** | 2, 311 | 0 (0, 0.05)^5^ | 114 | 0 (0, 0.04) | 115 | 0.716** |
| **Fermented cheese** | 2, 304 | 0.51 (0.12, 1.14) | 114 | 0.75 (0.22, 1.38) | 115 | 0.623** |
| **Fermented yoghurt and milk** | 2, 313 | 0.26 (0.07, 0.85) | 114 | 0.42 (0.1, 1.21) | 115 | 0.734** |
| **Fermented dairy products** | 2, 313 | 0.99 (0.35, 1.97) | 114 | 1.41 (0.64, 2.53) | 115 | 0.703** |
| **Fermented pulses and legumes** | 1, 521 | 0.07 (0.04, 0.21) | 71 | 0.07 (0.04, 0.12) | 63 | 0.817** |
| **Fermented meat or fish** | 1, 963 | 0.09 (0.05, 0.23) | 104 | 0.09 (0.05, 0.23) | 87 | 0.578** |
| **Fermented vegetables** | 2, 080 | 0.15 (0.09, 0.47) | 109 | 0.15 (0.09, 0.47) | 105 | 1.000** |
| **White or white sourdough bread** | 2, 073 | 0.42 (0.07, 0.85) | 106 | 0.53 (0.1, 1.02) | 103 | 0.658** |
| **Whole grain or whole grain sourdough bread** | 2, 090 | 0.26 (0.07, 0.85) | 109 | 0.42 (0.1, 1.02) | 103 | 0.672** |
| **Bread** | 2, 088 | 0.85 (0.36, 1.49) | 109 | 1.09 (0.49, 1.71) | 103 | 0.741** |
| **Fermented cereals (tarhana and all breads)** | 2, 134 | 0.9 (0.42, 1.54) | 108 | 1.09 (0.49, 1.72) | 103 | 0.740** |
| **Chocolate** | 1, 12t1 | 0.21 (0.07, 0.42) | 58 | 0.22 (0.09, 0.44) | 108 | 0.769** |
| **Fermented non-alcoholic soft beverages** | 1, 547 | 0.04 (0.02, 0.07) | 85 | 0.04 (0.02, 0.07) | 50 | 0.651** |
| **Vinegar-all types** | 1, 905 | 0.11 (0.05, 0.28) | 93 | 0.11 (0.06, 0.28) | 82 | 0.767** |
| **Coffee** | 765 | 1 (0.64, 1.05) | 30 | 1.02 (0.73, 1.09) | 96 | 0.510** |
| **Tea** | 1, 079 | 0.05 (0.02, 0.21) | 51 | 0.04 (0.02, 0.15) | 24 | 0.716** |
| **Cocoa beverages** | 1, 509 | 0.05 (0.02, 0.07) | 70 | 0.04 (0.02, 0.05) | 45 | 0.621** |
| **Beer or cider** | 1, 538 | 0.1 (0.05, 0.21) | 84 | 0.09 (0.05, 0.24) | 67 | 0.846** |
| **Wine-all types** | 1, 152 | 0.09 (0.05, 0.17) | 60 | 0.1 (0.06, 0.15) | 81 | 0.763** |
| **Strong spirits** | 1, 152 | 0.02 (0.02, 0.05) | 60 | 0.02 (0.02, 0.05) | 57 | 0.744** |

* p< 0.05, **p< 0.01; ^1^ 3FQ, Fermented Food Frequency Questionnaire; ^2^ n: study sample population.^3^ Spearman's correlation coefficient was used to determine the level of correlation between the 2 repetitions of the 3FQ. Intakes are presented as frequency/day and each main food group presented in this table sums the answers of the particular subgroups answered in the 3FQ. For example, food group “Fermented cheese” sums the consumptions that refer to the respective categories: “Hard Cheese (i.e. Parmigiano Reggiano, Grana Padano, Gruyere, Manchego, Cheddar, Pecorino)”, “Semi-hard cheese (i.e. Edam, Gouda)”, “Semi-hard cheese (i.e. Gorgonzola, Blue cheese)”, “Soft and/or Fresh cheese (i.e. Feta, Quark)” and “Soft and/or Fresh cheese (i.e. Chevre, Camembert, Brie)”; ^5^ Variables are presented as median (25th–75th percentile) following their skewed distribution.

Supplementary Table S4: Spearman’s correlation and ICC coefficients to check Repeatability of the 3FQ^1^ for Central-Eastern Europe

|  | **1st 3FQ^1^ n=5185)^2^** | | **2nd 3FQ (n=372)^2^** | | **r** | **Rs^3^** | **(ICC)^3^** |
| --- | --- | --- | --- | --- | --- | --- | --- |
|  | (n) | gr/day | (n) | gr/day |  |  |  |
| **Plant-based meat or dairy alternatives ^4^** | 5, 145 | 0 (0, 1.5)^5^ | 369 | 0 (0, 2.1)^5^ | 369 | 0.707** | 0.842** |
| **Fermented cheese** | 5, 101 | 5 (1, 19) | 367 | 13 (3.4, 26) | 363 | 0.590** | 0.623** |
| **Fermented yoghurt and milk** | 5, 166 | 45 (10, 136) | 371 | 64 (11, 163) | 370 | 0.753** | 0.735** |
| **Fermented dairy products** | 5, 178 | 67 (20, 169) | 372 | 80 (28, 196) | 371 | 0.732** | 0.734** |
| **Fermented pulses and legumes** | 2, 974 | 0.64 (0.10, 2.1) | 227 | 0.61 (0.10, 1.5) | 175 | 0.553** | 0.580** |
| **Fermented meat or fish** | 4, 266 | 3.4 (0.8, 10) | 323 | 3.4 (0.8, 5.6) | 286 | 0.681** | 0.791** |
| **Fermented vegetables** | 4, 722 | 7.5 (2, 20) | 345 | 8.4 (2.4, 20) | 335 | 0.636** | 0.638** |
| **White or white sourdough bread** | 4, 592 | 25 (3.2, 76) | 345 | 17 (2, 69) | 328 | 0.648** | 0.655** |
| **Whole grain or whole grain sourdough bread** | 4, 542 | 5.8 (0.79, 26) | 341 | 8.1 (1.3, 32) | 323 | 0.673** | 0.695** |
| **Bread** | 4, 621 | 42 (16, 106) | 345 | 42 (12, 109) | 328 | 0.598** | 0.626** |
| **Fermented cereals (trahana and all breads)** | 4, 647 | 44 (16, 111) | 345 | 42 (13, 110) | 330 | 0.606** | 0.627** |
| **Chocolate** | 4, 754 | 20 (5.7, 60) | 346 | 20 (6.6, 50) | 340 | 0.747** | 0.833** |
| **Fermented non-alcoholic soft beverages** | 1, 209 | 3.6 (1.2, 8.9) | 86 | 3.6 (1.2, 8.9) | 65 | 0.778** | **0.329** |
| **Vinegar-all types** | 3, 193 | 2.3 (0.39, 9.5) | 281 | 2.6 (0.59, 8.9) | 252 | 0.687** | 0.803** |
| **Coffee** | 4, 209 | 60 (20, 150) | 322 | 60 (30, 153) | 311 | 0.510** | 0.645** |
| **Tea** | 2, 177 | 2.4 (0, 21) | 164 | 0 (0, 12) | 121 | 0.604** | 0.909** |
| **Cocoa beverages** | 2, 434 | 9.5 (4.7, 25) | 213 | 9.5 (4.7, 24) | 183 | 0.583** | 0.569** |
| **Beer or cider** | 2, 886 | 25 (10, 76) | 242 | 27 (10, 86) | 224 | 0.782** | 0.737** |
| **Wine-all types** | 3, 333 | 13 (5.8, 31) | 283 | 15 (7.5, 51) | 270 | 0.743** | 0.963** |
| **Strong spirits** | 2, 648 | 5.9 (2.4, 25) | 225 | 5.9 (2.4, 25) | 210 | 0.735** | 0.789** |

* p< 0.05, **p< 0.01; ^1^ 3FQ, Fermented Food Frequency Questionnaire; ^2^ n: study sample population.;^3^ Spearman's correlation coefficient was used to determine the level of correlation between the 2 repetitions of the 3FQ. ^3^Additionally, Intraclass Correlation Coefficient (ICC) was used to determine intra-class correlation between them. Spearman's correlation coefficient is interpreted as follows 0.1 - 0.3: weak correlation, 0.3-0.5: medium correlation and 0.5-1.0 strong correlation. ICC is interpreted respectively: ICC<0.5 poor, 0.5≤ICC<0.75 moderate, 0.75≤ICC<0.9 good and ICC≥0.9 excellent reliability.; ^4^ Intakes are presented as g/day and each main food group presented in this table sums the answers of the subgroups answered in the 3FQ. For example, food group “Fermented cheese” sums the consumptions that refer to the respective categories: “Hard Cheese (i.e. Parmigiano Reggiano, Grana Padano, Gruyere, Manchego, Cheddar, Pecorino)”, “Semi-hard cheese (i.e. Edam, Gouda)”, “Semi-hard cheese (i.e. Gorgonzola, Blue cheese)”, “Soft and/or Fresh cheese (i.e. Feta, Quark)” and “Soft and/or Fresh cheese (i.e. Chevre, Camembert, Brie)”; ^5^ Variables are presented as median (25th–75th percentile) following their skewed distribution.

Supplementary Table S5: Spearman’s correlation to check Repeatability of the 3FQ^1^ for Central-Eastern Europe for frequency/day

|  | **1st 3FQ (n=5185)^2^** | | **2nd 3FQ (n=372)^2^** | | **r** | **Rs^3^** |
| --- | --- | --- | --- | --- | --- | --- |
|  | (n) | frequency/day | (n) | frequency/day |  |  |
| **Plant-based meat or dairy aletrnatives ^4^** | 5, 173 | 0 (0, 0.02)^5^ | 372 | 0 (0, 0.04) | 372 | 0.712** |
| **Fermented cheese** | 5, 166 | 0.26 (0.06, 0.73) | 372 | 0.57 (0.21, 1) | 371 | 0.646** |
| **Fermented yoghurt and milk** | 5, 173 | 0.26 (0.05, 0.69) | 371 | 0.42 (0.07, 0.85) | 370 | 0.760** |
| **Fermented dairy products** | 5, 180 | 0.85 (0.26, 1.5) | 372 | 1.1 (0.42, 1.8) | 371 | 0.698** |
| **Fermented pulses and legumes** | 3, 063 | 0.05 (0.02, 0.09) | 228 | 0.05 (0.02, 0.09) | 178 | 0.624** |
| **Fermented meat or fish** | 4, 734 | 0.21 (0.05, 0.64) | 346 | 0.21 (0.05, 0.26) | 290 | 0.710** |
| **Fermented vegetables** | 4, 620 | 0.26 (0.1, 0.64) | 345 | 0.26 (0.1, 0.64) | 347 | 1.000** |
| **White or white sourdough bread** | 4, 606 | 0.64 (0.07, 1) | 343 | 0.26 (0.04, 1) | 331 | 0.769** |
| **Whole grain or whole grain sourdough bread** | 4, 656 | 0.1 (0.02, 0.42) | 345 | 0.21 (0.04, 0.42) | 331 | 0.725** |
| **Bread** | 4, 603 | 0.85 (0.33, 1.1) | 341 | 0.73 (0.27, 1) | 331 | 0.671** |
| **Fermented cereals (trahana and all breads)** | 4, 725 | 0.87 (0.42, 1.1) | 343 | 0.75 (0.28, 1.1) | 331 | 0.668** |
| **Chocolate** | 1, 196 | 0.23 (0.09, 0.64) | 85 | 0.25 (0.1, 0.64) | 340 | 0.727** |
| **Fermented non-alcoholic soft beverages** | 3, 179 | 0.02 (0.02, 0.05) | 276 | 0.02 (0.02, 0.06) | 66 | 0.704** |
| **Vinegar-all types** | 4, 133 | 0.23 (0.07, 0.68) | 315 | 0.27 (0.09, 0.71) | 253 | 0.701** |
| **Coffee** | 2, 105 | 1 (0.64, 1.1) | 164 | 1 (0.89, 1.2) | 315 | 0.550** |
| **Tea** | 2, 389 | 0.02 (0, 0.12) | 210 | 0 (0, 0.05) | 126 | 0.582** |
| **Cocoa beverages** | 2, 880 | 0.05 (0.02, 0.21) | 242 | 0.05 (0.02, 0.1) | 183 | 0.569** |
| **Beer or cider** | 3, 319 | 0.07 (0.045, 0.21) | 281 | 0.07 (0.05, 0.21) | 224 | 0.731** |
| **Wine-all types** | 2, 652 | 0.07 (0.05, 0.17) | 225 | 0.1 (0.05, 0.21) | 272 | 0.695** |
| **Strong spirits** | 2, 652 | 0.05 (0.02, 0.05) | 225 | 0.05 (0.02, 0.05) | 210 | 0.668** |

* p< 0.05, **p< 0.01; ^1^ 3FQ, Fermented Food Frequency Questionnaire; ^2^ n: study sample population.^3^ Spearman's correlation coefficient was used to determine the level of correlation between the 2 repetitions of the 3FQ. Intakes are presented as frequency/day and each main food group presented in this table sums the answers of the subgroups answered in the 3FQ. For example, food group “Fermented cheese” sums the consumptions that refer to the respective categories: “Hard Cheese (i.e. Parmigiano Reggiano, Grana Padano, Gruyere, Manchego, Cheddar, Pecorino)”, “Semi-hard cheese (i.e. Edam, Gouda)”, “Semi-hard cheese (i.e. Gorgonzola, Blue cheese)”, “Soft and/or Fresh cheese (i.e. Feta, Quark)” and “Soft and/or Fresh cheese (i.e. Chevre, Camembert, Brie)”; ^5^ Variables are presented as median (25th–75th percentile) following their skewed distribution.

Supplementary Table S6: Spearman’s correlation and ICC coefficients to check Repeatability of the 3FQ^1^ for Western Europe

|  | **1st 3FQ^1^ n=2577)^2^** | | **2nd 3FQ (n=338)^2^** | | **r** | **Rs^3^** | **(ICC)^3^** |
| --- | --- | --- | --- | --- | --- | --- | --- |
|  | (n) | gr/day | (n) | gr/day |  |  |  |
| **Plant-based meat or dairy alternatives ^4^** | 2, 569 | 3.6 (0, 16)^5^ | 338 | 3.8 (0, 15)^5^ | 338 | 0.752** | 0.716** |
| **Fermented cheese** | 2, 567 | 20 (6.9, 41) | 338 | 25 (9.5, 46) | 338 | 0.675** | 0.788** |
| **Fermented yoghurt and milk** | 2, 569 | 44 (10, 128) | 338 | 44 (10, 128) | 338 | 0.805** | 0.796** |
| **Fermented dairy products** | 2, 573 | 80 (32, 174) | 338 | 92 (35, 170) | 338 | 0.816** | 0.800** |
| **Fermented pulses and legumes** | 2, 059 | 1.3 (0.3, 3.7) | 279 | 1.3 (0.34, 3.8) | 258 | 0.672** | 0.812** |
| **Fermented meat or fish** | 1, 939 | 1.3 (0.8, 3.7) | 272 | 1.3 (0.8, 4) | 254 | 0.623** | 0.807** |
| **Fermented vegetables** | 2, 379 | 4.4 (2, 9.6) | 325 | 4.8 (2, 9.8) | 311 | 0.682** | 0.707** |
| **White or white sourdough bread** | 2, 418 | 17 (3.2, 65) | 318 | 19 (2.7, 75) | 311 | 0.668** | 0.785** |
| **Whole grain or whole grain sourdough bread** | 2, 409 | 29 (6.7, 91) | 317 | 32 (7.6, 98) | 309 | 0.732** | 0.773** |
| **Bread** | 2, 435 | 72 (25, 165) | 318 | 89 (27, 171) | 313 | 0.697** | 0.792** |
| **Fermented cereals (tarhana and all breads)** | 2, 460 | 71 (25, 164) | 320 | 88 (27, 170) | 315 | 0.678** | 0.786** |
| **Chocolate** | 2, 470 | 20 (6.6, 47) | 331 | 22 (9.9, 47) | 327 | 0.618** | 0.827** |
| **Fermented non-alcoholic soft beverages** | 774 | 7.1 (3.6, 19) | 115 | 8 (3.6, 20) | 102 | 0.781** | 0.939** |
| **Vinegar-all types** | 2, 090 | 3.4 (1, 9.9) | 286 | 4.4 (1.3, 11) | 268 | 0.699** | 0.829** |
| **Coffee** | 2, 001 | 66 (30, 240) | 261 | 80 (25, 240) | 254 | 0.710** | 0.846** |
| **Tea** | 1, 093 | 0 (0, 14) | 136 | 0 (0, 14) | 107 | 0.768** | 0.764** |
| **Cocoa beverages** | 1, 308 | 9.5 (4.7, 17) | 181 | 9.5 (4.7, 17) | 157 | 0.604** | 0.870** |
| **Beer or cider** | 1, 957 | 50 (23, 130) | 257 | 60 (25, 140) | 250 | 0.853** | 0.855** |
| **Wine-all types** | 1, 948 | 25 (10, 61) | 259 | 41 (15, 90) | 253 | 0.823** | 0.716** |
| **Strong spirits** | 1, 376 | 3.5 (2.4, 8.9) | 173 | 3.5 (2.4, 5.9) | 155 | 0.656** | 0.728** |

* p< 0.05, **p< 0.01; ^1^ 3FQ, Fermented Food Frequency Questionnaire; ^2^ n: study sample population.;^3^ Spearman's correlation coefficient was used to determine the level of correlation between the 2 repetitions of the 3FQ. ^3^Additionally, Intraclass Correlation Coefficient (ICC) was used to determine intra-class correlation between them. Spearman's correlation coefficient is interpreted as follows 0.1 - 0.3: weak correlation, 0.3-0.5: medium correlation and 0.5-1.0 strong correlation. ICC is interpreted respectively: ICC<0.5 poor, 0.5≤ICC<0.75 moderate, 0.75≤ICC<0.9 good and ICC≥0.9 excellent reliability.; ^4^ Intakes are presented as g/day and each main food group presented in this table sums the answers of the particular subgroups answered in the 3FQ. For example, food group “Fermented cheese” sums the consumptions that refer to the respective categories: “Hard Cheese (i.e. Parmigiano Reggiano, Grana Padano, Gruyere, Manchego, Cheddar, Pecorino)”, “Semi-hard cheese (i.e. Edam, Gouda)”, “Semi-hard cheese (i.e. Gorgonzola, Blue cheese)”, “Soft and/or Fresh cheese (i.e. Feta, Quark)” and “Soft and/or Fresh cheese (i.e. Chevre, Camembert, Brie)”; ^5^ Variables are presented as median (25th–75th percentile) following their skewed distribution.

Supplementary Table S7: Spearman’s correlation to check Repeatability of the 3FQ^1^ for Western Europe for frequency/day

|  | **1st 3FQ (n=2577)^2^** | | **2nd 3FQ (n=338)^2^** | | **r** | **Rs^3^** |
| --- | --- | --- | --- | --- | --- | --- |
|  | (n) | frequency/day | (n) | frequency/day |  |  |
| **Plant-based meat or dairy alternatives ^4^** | 2, 574 | 0.04 (0, 0.21)^5^ | 338 | 0.04 (0, 0.12) | 338 | 0.787** |
| **Fermented cheese** | 2, 573 | 0.79 (0.33, 1.4) | 338 | 0.85 (0.41, 1.5) | 338 | 0.658** |
| **Fermented yoghurt and milk** | 2, 572 | 0.23 (0.05, 0.69) | 338 | 0.23 (0.05, 0.85) | 338 | 0.819** |
| **Fermented dairy products** | 2, 573 | 1.3 (0.68, 2) | 338 | 1.4 (0.7, 2.2) | 338 | 0.712** |
| **Fermented pulses and legumes** | 2, 072 | 0.07 (0.04, 0.23) | 279 | 0.07 (0.05, 0.23) | 260 | 0.683** |
| **Fermented meat or fish** | 2, 376 | 0.09 (0.05, 0.23) | 325 | 0.07 (0.05, 0.23) | 255 | 0.627** |
| **Fermented vegetables** | 2, 439 | 0.12 (0.07, 0.28) | 318 | 0.12 (0.07, 0.28) | 325 | 1.000** |
| **White or white sourdough bread** | 2, 445 | 0.21 (0.04, 0.64) | 316 | 0.23 (0.04, 0.66) | 315 | 0.677** |
| **Whole grain or whole grain sourdough bread** | 2, 468 | 0.26 (0.07, 0.69) | 320 | 0.26 (0.07, 0.85) | 315 | 0.738** |
| **Bread** | 2, 454 | 0.73 (0.3, 1.1) | 317 | 0.84 (0.36, 1.1) | 315 | 0.688** |
| **Fermented cereals (tarhana and all breads)** | 2, 438 | 0.73 (0.3, 1.1) | 328 | 0.84 (0.36, 1.1) | 315 | 0.690** |
| **Chocolate** | 764 | 0.28 (0.12, 0.69) | 116 | 0.28 (0.21, 0.69) | 327 | 0.649** |
| **Fermented non-alcoholic soft beverages** | 2, 053 | 0.05 (0.02, 0.1) | 283 | 0.05 (0.02, 0.1) | 104 | 0.716** |
| **Vinegar-all types** | 1, 969 | 0.31 (0.14, 0.7) | 260 | 0.44 (0.2, 0.84) | 269 | 0.758** |
| **Coffee** | 1, 077 | 1 (0.66, 1) | 134 | 1 (0.68, 1.1) | 256 | 0.703** |
| **Tea** | 1, 290 | 0 (0, 0.05) | 179 | 0 (0, 0.05) | 107 | 0.741** |
| **Cocoa beverages** | 1, 954 | 0.04 (0.02, 0.07) | 256 | 0.04 (0.02, 0.07) | 157 | 0.624** |
| **Beer or cider** | 1, 942 | 0.11 (0.06, 0.25) | 259 | 0.12 (0.07, 0.28) | 250 | 0.858** |
| **Wine-all types** | 1, 378 | 0.12 (0.07, 0.27) | 173 | 0.14 (0.09, 0.44) | 253 | 0.841** |
| **Strong spirits** | 1, 378 | 0.02 (0.02, 0.05) | 173 | 0.02 (0.02, 0.05) | 155 | 0.677** |

* p< 0.05, **p< 0.01; ^1^ 3FQ, Fermented Food Frequency Questionnaire; ^2^ n: study sample population.^3^ Spearman's correlation coefficient was used to determine the level of correlation between the 2 repetitions of the 3FQ. Intakes are presented as frequency/day and each main food group presented in this table sums the answers of the subgroups answered in the 3FQ. For example, food group “Fermented cheese” sums the consumptions that refer to the respective categories: “Hard Cheese (i.e. Parmigiano Reggiano, Grana Padano, Gruyere, Manchego, Cheddar, Pecorino)”, “Semi-hard cheese (i.e. Edam, Gouda)”, “Semi-hard cheese (i.e. Gorgonzola, Blue cheese)”, “Soft and/or Fresh cheese (i.e. Feta, Quark)” and “Soft and/or Fresh cheese (i.e. Chevre, Camembert, Brie)”; ^5^ Variables are presented as median (25th–75th percentile) following their skewed distribution.

Supplementary Table S8: Spearman’s correlation and ICC coefficients to check Repeatability of the 3FQ^1^ for Southern Europe

|  | **1st 3FQ^1^ n=2569)^2^** | | **2nd 3FQ (n=424)^2^** | | **r** | **Rs^3^** | **(ICC)^3^** |
| --- | --- | --- | --- | --- | --- | --- | --- |
|  | (n) | gr/day | (n) | gr/day |  |  |  |
| **Plant-based meat or dairy alternatives ^4^** | 2, 557 | 0 (0, 2.8)^5^ | 424 | 0 (0, 2.5)^5^ | 423 | 0.758** | 0.757** |
| **Fermented cheese** | 2, 540 | 13 (2.6, 26) | 422 | 13 (4.2, 29) | 422 | 0.557** | 0.603** |
| **Fermented yoghurt and milk** | 2, 566 | 56 (10, 130) | 424 | 57 (10, 133) | 424 | 0.639** | 0.762** |
| **Fermented dairy products** | 2, 569 | 78 (27, 163) | 424 | 79 (29, 171) | 424 | 0.613** | 0.768** |
| **Fermented pulses and legumes** | 1, 697 | 0.84 (0.10, 3.7) | 246 | 0.74 (0.10, 2.6) | 201 | 0.535** | 0.666** |
| **Fermented meat or fish** | 1, 590 | 0.8 (0.32, 3.4) | 241 | 0.9 (0.32, 3.4) | 196 | 0.543** | 0.528** |
| **Fermented vegetables** | 2, 153 | 8.4 (2, 22) | 360 | 8.8 (2.3, 26) | 331 | 0.706** | 0.823** |
| **White or white sourdough bread** | 2, 388 | 36 (7.3, 94) | 406 | 52 (13, 108) | 391 | 0.599** | 0.721** |
| **Whole grain or whole grain sourdough bread** | 2, 364 | 7.7 (0.86, 36) | 404 | 4.7 (0, 32) | 387 | 0.719** | 0.699** |
| **Bread** | 2, 394 | 61 (25, 130) | 406 | 74 (36, 148) | 392 | 0.549** | 0.710** |
| **Fermented cereals (trahana and all breads)** | 2, 409 | 65 (26, 134) | 407 | 77 (38, 154) | 393 | 0.556** | 0.709** |
| **Chocolate** | 2, 336 | 12 (4.7, 37) | 379 | 12 (4.2, 33) | 354 | 0.640** | 0.541** |
| **Fermented non-alcoholic soft beverages** | 466 | 2.7 (0, 8.9) | 68 | 3.6 (1.2, 8.9) | 45 | 0.818** | 0.513* |
| **Vinegar-all types** | 1, 680 | 2.1 (0.49, 6.3) | 261 | 2.5 (0.59, 6.6) | 231 | 0.685** | 0.865** |
| **Coffee** | 2, 100 | 60 (14, 156) | 338 | 60 (19, 150) | 324 | 0.489** | 0.674** |
| **Tea** | 963 | 17 (0, 237) | 146 | 27 (0, 379) | 97 | 0.847** | 0.864** |
| **Cocoa beverages** | 949 | 9.5 (4.7, 24) | 150 | 9.5 (4.7, 17) | 118 | 0.668** | 0.492** |
| **Beer or cider** | 1, 302 | 33 (17, 102) | 208 | 35 (17, 105) | 194 | 0.713** | 0.838** |
| **Wine-all types** | 1, 319 | 19 (7.5, 56) | 200 | 23 (11, 60) | 190 | 0.690** | 0.900** |
| **Strong spirits** | 900 | 5.9 (2.4, 18) | 143 | 5.9 (2.4, 8.9) | 122 | 0.668** | 0.739** |

* p< 0.05, **p< 0.01; ^1^ 3FQ, Fermented Food Frequency Questionnaire; ^2^ n: study sample population.;^3^ Spearman's correlation coefficient was used to determine the level of correlation between the 2 repetitions of the 3FQ. ^3^Additionally, Intraclass Correlation Coefficient (ICC) was used to determine intra-class correlation between them. Spearman's correlation coefficient is interpreted as follows 0.1 - 0.3: weak correlation, 0.3-0.5: medium correlation and 0.5-1.0 strong correlation. ICC is interpreted respectively: ICC<0.5 poor, 0.5≤ICC<0.75 moderate, 0.75≤ICC<0.9 good and ICC≥0.9 excellent reliability.; ^4^ Intakes are presented as g/day and each main food group presented in this table sums the answers of the particular subgroups answered in the 3FQ. For example, food group “Fermented cheese” sums the consumptions that refer to the respective categories: “Hard Cheese (i.e. Parmigiano Reggiano, Grana Padano, Gruyere, Manchego, Cheddar, Pecorino)”, “Semi-hard cheese (i.e. Edam, Gouda)”, “Semi-hard cheese (i.e. Gorgonzola, Blue cheese)”, “Soft and/or Fresh cheese (i.e. Feta, Quark)” and “Soft and/or Fresh cheese (i.e. Chevre, Camembert, Brie)”; ^5^ Variables are presented as median (25th–75th percentile) following their skewed distribution.

Supplementary Table S9: Spearman’s correlation to check Repeatability of the 3FQ^1^ for Southern Europe for frequency/day

|  | **1st 3FQ (n=2569)^2^** | | **2nd 3FQ (n=424)^2^** | | **r** | **Rs^3^** |
| --- | --- | --- | --- | --- | --- | --- |
|  | (n) | frequency/day | (n) | frequency/day |  |  |
| **Plant-based meat or dairy alternatives ^4^** | 2, 564 | 0 (0, 0.04)^5^ | 424 | 0 (0, 0.06) | 424 | 0.751** |
| **Fermented cheese** | 2, 562 | 0.6 (0.21, 1) | 424 | 0.67 (0.21, 1.1) | 424 | 0.600** |
| **Fermented yoghurt and milk** | 2, 568 | 0.42 (0.07, 0.85) | 424 | 0.42 (0.07, 0.85) | 424 | 0.642** |
| **Fermented dairy products** | 2, 569 | 1 (0.46, 1.8) | 424 | 1.2 (0.61, 2) | 424 | 0.597** |
| **Fermented pulses and legumes** | 1, 736 | 0.05 (0.02, 0.21) | 247 | 0.05 (0.02, 0.15) | 209 | 0.620** |
| **Fermented meat or fish** | 2, 152 | 0.05 (0.02, 0.21) | 360 | 0.09 (0.05, 0.23) | 201 | 0.528** |
| **Fermented vegetables** | 2, 389 | 0.26 (0.1, 0.9) | 405 | 0.23 (0.09, 0.63) | 360 | 1.000** |
| **White or white sourdough bread** | 2, 372 | 0.64 (0.21, 1) | 404 | 0.42 (0.07, 1) | 393 | 0.597** |
| **Whole grain or whole grain sourdough bread** | 2, 413 | 0.1 (0.02, 0.64) | 407 | 0.21 (0.04, 0.66) | 392 | 0.781** |
| **Bread** | 2, 400 | 1 (0.46, 1.2) | 407 | 0.89 (0.44, 1.1) | 393 | 0.465** |
| **Fermented cereals (trahana and all breads)** | 2, 301 | 1 (0.49, 1.3) | 376 | 0.92 (0.46, 1.2) | 393 | 0.499** |
| **Chocolate** | 460 | 0.21 (0.07, 0.64) | 68 | 0.25 (0.1, 0.66) | 356 | 0.679** |
| **Fermented non-alcoholic soft beverages** | 1, 642 | 0.02 (0.02, 0.05) | 255 | 0.04 (0.02, 0.07) | 45 | 0.742** |
| **Vinegar-all types** | 2, 057 | 0.23 (0.08, 0.65) | 331 | 0.28 (0.1, 0.73) | 231 | 0.661** |
| **Coffee** | 934 | 1 (0.42, 1.1) | 145 | 1 (0.68, 1.1) | 329 | 0.701** |
| **Tea** | 920 | 0.06 (0, 1) | 147 | 0.02 (0, 0.15) | 97 | 0.790** |
| **Cocoa beverages** | 1, 298 | 0.05 (0.02, 0.1) | 207 | 0.05 (0.02, 0.07) | 118 | 0.595** |
| **Beer or cider** | 1, 301 | 0.08 (0.05, 0.21) | 200 | 0.09 (0.05, 0.23) | 67 | 0.846** |
| **Wine-all types** | 901 | 0.1 (0.06, 0.25) | 144 | 0.12 (0.06, 0.28) | 191 | 0.741** |
| **Strong spirits** | 901 | 0.02 (0.02, 0.05) | 144 | 0.02 (0.02, 0.05) | 122 | 0.669** |

* p< 0.05, **p< 0.01; ^1^ 3FQ, Fermented Food Frequency Questionnaire; ^2^ n: study sample population.^3^ Spearman's correlation coefficient was used to determine the level of correlation between the 2 repetitions of the 3FQ. Intakes are presented as frequency/day and each main food group presented in this table sums the answers of the subgroups answered in the 3FQ. For example, food group “Fermented cheese” sums the consumptions that refer to the respective categories: “Hard Cheese (i.e. Parmigiano Reggiano, Grana Padano, Gruyere, Manchego, Cheddar, Pecorino)”, “Semi-hard cheese (i.e. Edam, Gouda)”, “Semi-hard cheese (i.e. Gorgonzola, Blue cheese)”, “Soft and/or Fresh cheese (i.e. Feta, Quark)” and “Soft and/or Fresh cheese (i.e. Chevre, Camembert, Brie)”; ^5^ Variables are presented as median (25th–75th percentile) following their skewed distribution.

Supplementary Table S10: %Within the Agreement Interval & Spearman’s correlation coefficients to check Validity of the 3FQ^1^ on the whole Sample using 24-hour dietary Recalls (All food groups)

|  | **Consumption as grams per day** | | | | | |
| --- | --- | --- | --- | --- | --- | --- |
|  | **3FQ (n=391)** | **24hR** | **24hR- Daily consumption (n=391)^2^** | **Spearman Correlation**  **n** | **% Within the Agreement Interval^3^** | **Spearman Correlation**  **Coefficient^4^** |
| **Plant-based meat or dairy alternative** ^5^ | 0 (0, 0.82) ^6^ | 10 | 20.25 (11.05, 60) | 10 | 90 | 0.649* |
| **Fermented dairy products** | 90.84 (41.38, 193.95) | 329 | 125 (42.9, 264.94) | 329 | 93.92 | 0.244** |
| **Fermented cheese** |  |  |  |  |  |  |
| Hard cheese(i.e. Parmigiano Reggiano) | 1.95 (0.4, 4.2) | 94 | 25.14 (10, 39.8) | 94 | 96.81 | 0.033 |
| Semi-hard cheese (i.e. Edam) | 4.2 (1, 12.8) | 149 | 22.03 (12.5, 49.75) | 118 | 95.76 | 0.089 |
| Semi-hard cheese(i.e. Gorgonzola) | 0.4 (0, 1) | 9 | 44.69 (32.95, 80.6) | 7 | 100 | 0.356 |
| Soft and/or Fresh cheese (i.e. Feta) | 4.2 (1, 12.8) | 90 | 31.38 (19.21, 55.75) | 70 | 95.71 | 0.249* |
| Soft and/or Fresh cheese (i.e. Chèvre) | 0.4 (0, 1) | 72 | 30 (17.74, 50) | 52 | 94.23 | 0.213 |
| **Fermented yoghurt and milk** | 12.8 (4.2, 31.56) | 257 | 44 (24.36, 85.58) | 256 | 94.53 | 0.136* |
| Fermented Yoghurt | 42 (10, 128) | 157 | 200 (100, 400) | 157 | 96.18 | 0.200* |
| Fermented milk | 2.5 (0, 21) | 51 | 100 (40, 200) | 36 | 94.44 | 0.194 |
|  | 64 (21, 149) | 188 | 200 (100, 364.39) | 188 | 95.21 | 0.161* |
| **Fermented pulses and legumes** | 0.61 (0.10, 2.62) | 17 | 10 (5.65, 41) | 16 | 93.75 | 0.461 |
| **Fermented meat or fish** | 1.3 (0.47, 5.46) | 11 | 52 (34.62, 80) | 10 | 90 | 0.39 |
| Olives | 2 (0.8, 8.4) | 33 | 15 (10, 20) | 30 | 96.67 | 0.113 |
| Fermented cabbage (i.e. Sauerkraut) | 0.5 (0.2, 3.53) | 40 | 97 (52.29, 137.81) | 26 | 96.15 | 0.29 |
| Other fermented vegetables | 0.695 (0, 3.53) | 22 | 38 (22.5, 60) | 19 | 89.47 | 0.258 |
| **Fermented vegetables** | 4.79 (1.38, 14.49) | 87 | 40 (20, 97) | 83 | 95.18 | 0.022 |
| White bread | 15.15 (1.51, 55.50) | 198 | 52.5 (32, 90) | 183 | 93.44 | 0.081 |
| White sourdough bread | 2.51 (0.40, 18.84) | 22 | 25.25 (12.5, 36.75) | 21 | 95.24 | 0.418 |
| **Fermented grains/cereals (tarhana and all breads)** | 59.81 (18.47, 130.25) | 283 | 50 (28.6, 87.4) | 164 | 95.73 | 0.139 |
| **Bread** | 59.57 (17.49, 129.35) | 283 | 61 (36, 95) | 271 | 94.83 | 0.095 |
| **White or white sourdough bread** | 25.42 (3.02, 79.43) | 210 | 52 (32, 87.29) | 198 | 93.43 | 0.146* |
| **Whole grain or whole grain sourdough bread** | 11.63 (1.97, 51.28) | 121 | 47.5 (30, 76.75) | 119 | 94.12 | 0.128 |
| Whole grain bread | 7.50 (0.94, 43.08) | 111 | 48 (30, 76.75) | 107 | 95.33 | 0.093 |
| Whole grain sourdough bread | 1.47 (0, 9.88) | 17 | 23 (17.63, 36) | 15 | 96.67 | 0.391 |
| Tarhana | 0 (0, 0.7) | 5 | 192 ± 75.40 | 5 | 100 | 0.616 |
| **Chocolate** | 16.05 (4.72, 40.59) | 105 | 20 (10, 40) | 103 | 96.12 | 0.152 |
| Milk chocolate | 4.72 (0.94, 19.82) | 65 | 20.25 (12, 45) | 62 | 95.16 | 0.334* |
| Dark chocolate | 4.72 (0.94, 19.82) | 43 | 15 (7.5, 25) | 33 | 96.97 | 0.249 |
| White chocolate | 0.94 (0, 1.89) | 2 | 35.42 ± 2.95 | 2 | 100 | 1 |
| **Fermented non-alcoholic beverages** | 3.56 (1.78, 10.09) | 31 | 180 (125, 250) | 15 | 93.33 | 0.369 |
| **Vinegar-all types** | 2.88 (0.54, 9.69) | 82 | 4.6 (2.47, 7.29) | 68 | 91.18 | 0.015 |
| Apple vinegar | 0.25 (0.10, 3.10) | 28 | 3.12 (2.06, 6.21) | 22 | 95.45 | 0.035 |
| Grape vinegar | 0.25 (0, 1.03) | 7 | 2.39 (1.11, 6.59) | 5 | 100 | 0.359 |
| Balsamic vinegar | 0.30 (0.10, 3.10) | 32 | 4.97 (2.27, 11.64) | 24 | 95.83 | 0.041 |
| Other vinegar (*i.e*. Rice vinegar) | 0.10 (0, 0.25) | 22 | 4.6 (2.65, 6.9) | 12 | 91.67 | 0.287 |
| **Coffee** | 65.94 (30, 169.59) | 266 | 227 (115, 377) | 255 | 92.94 | 0.233** |
| Espresso coffee | 30 (1.5, 60) | 167 | 155.5 (80, 276.5) | 162 | 96.3 | 0.157* |
| Arabic coffee | 1.2 (0, 19.2) | 39 | 222 (100, 300) | 27 | 96.3 | 0.546* |
| Filter coffee | 4.74 (0, 18.96) | 27 | 237 (126, 303) | 22 | 90.91 | 0.226 |
| Instant coffee | 2.37 (0, 29.63) | 81 | 250 (126, 267) | 46 | 95.65 | 0.242 |
| **Cocoa beverages** | 9.48 (4.74, 16.59) | 21 | 4.23 (2.11, 250) | 14 | 100 | 0.094 |
| **Beer or cider** | 35 (16.5, 90.75) | 30 | 261 (165, 500) | 26 | 92.31 | 0.338 |
| **Wine-all types** | 26.25 (7.5, 61.25) | 55 | 150 (96, 200) | 51 | 94.12 | 0.277* |
| **Strong spirits** | 5.92 (3.55, 17.75) | 13 | 50 (29.5, 65) | 9 | 88.89 | 0.311 |

* p< 0.05, **p< 0.01; ^1^ 3FQ: Fermented Food Frequency Questionnaire. ^2^N: study sample population used for the validation analysis.^3^ % within the agreement interval between the results of the FPQ and the 24hR per food group. Bland and Altman analysis was used to determine the percentage of agreement between the two methods.^4^ Spearman's correlation coefficient was used to determine the level of correlation between the 2 repetitions of the 3FQ. Spearman's correlation coefficient is interpreted as follows 0.1 - 0.3: weak correlation, 0.3-0.5: medium correlation and 0.5-1.0 strong correlation).^5^ Intakes are presented as g/day. ^6^ Variables are presented as median (25th–75th percentile) following their skewed distribution, except for tarhana food group which is normally distributed and presented as mean ± SD.


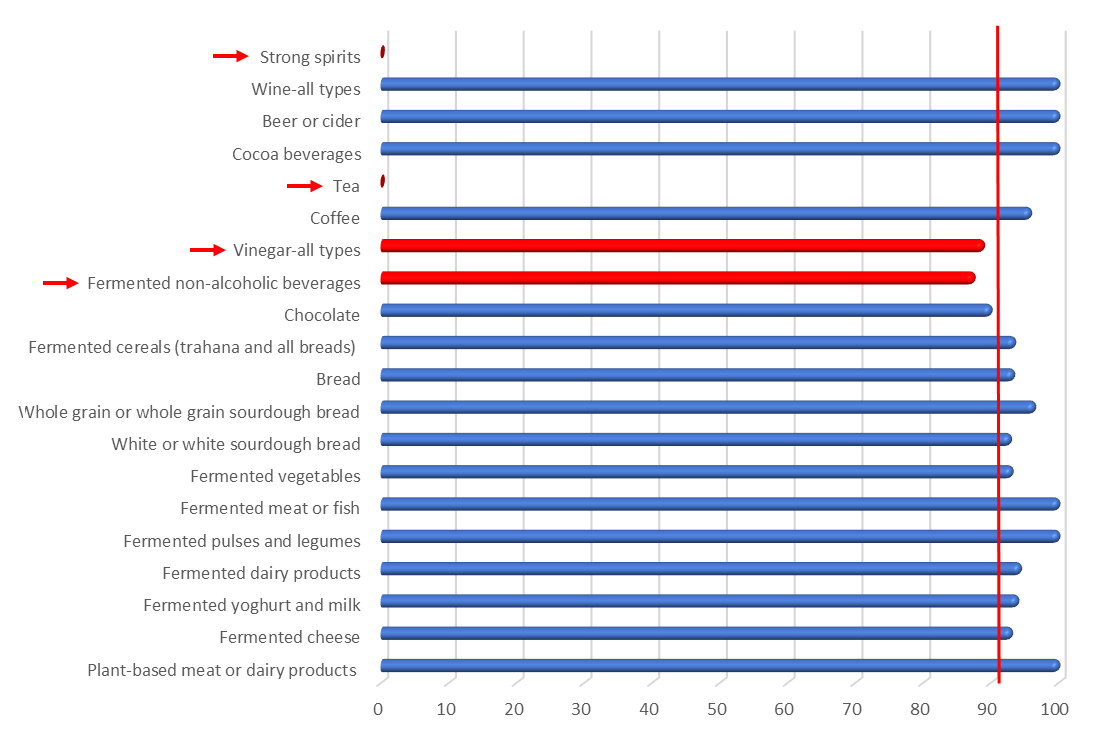
Figure

Supplementary Figure S1: Percent (%) within the agreement interval to assess the validity of the 3FQ^1,2,3^ for Northern Europe**Correlation is significant at the 0.001 level (2-tailed).^1^ 3FQ: Fermented Food Frequency Questionnaire .^2^ N=391 (validity study sample population).^3^ % within the agreement interval between the results of the 3FQ and the 24hR per food group. Bland and Altman analysis was used to determine the percentage of agreement between the two methods. Red bars indicate the food subcategories that an agreement was not obtained through Bland-Altman analysis.


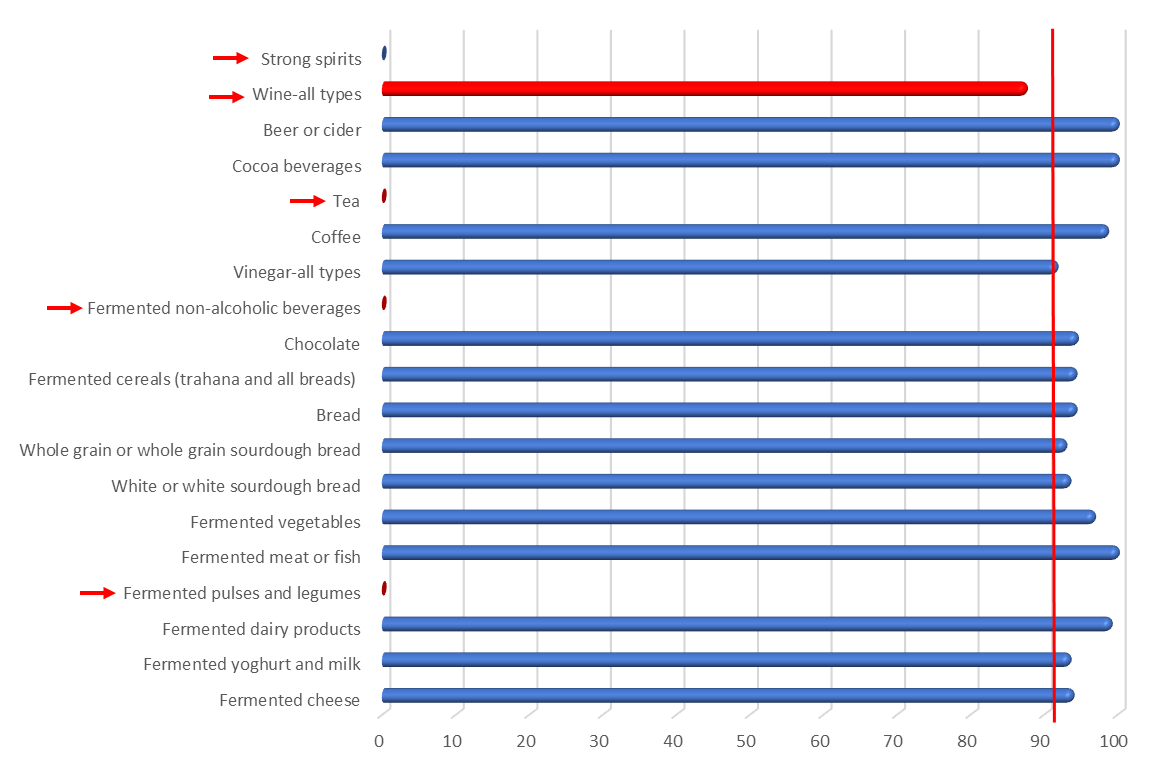


Supplementary Figure S 2. % within the agreement interval to assess the validity of the 3FQ^1,2,3^ for Central-Eastern *Correlation is significant at the 0.05 level (2-tailed), **Correlation is significant at the 0.001 level (2-tailed).1 3FQ: Fermented Food Frequency Questionnaire.2 n=391 (validity study sample population).3 % within the agreement interval between the results of the 3FQ and the 24hR per food group. Bland and Altman analysis was used to determine the percentage of agreement between the two methods. Red bars indicate the food subcategories that an agreement was not obtained through Bland-Altman analysis.


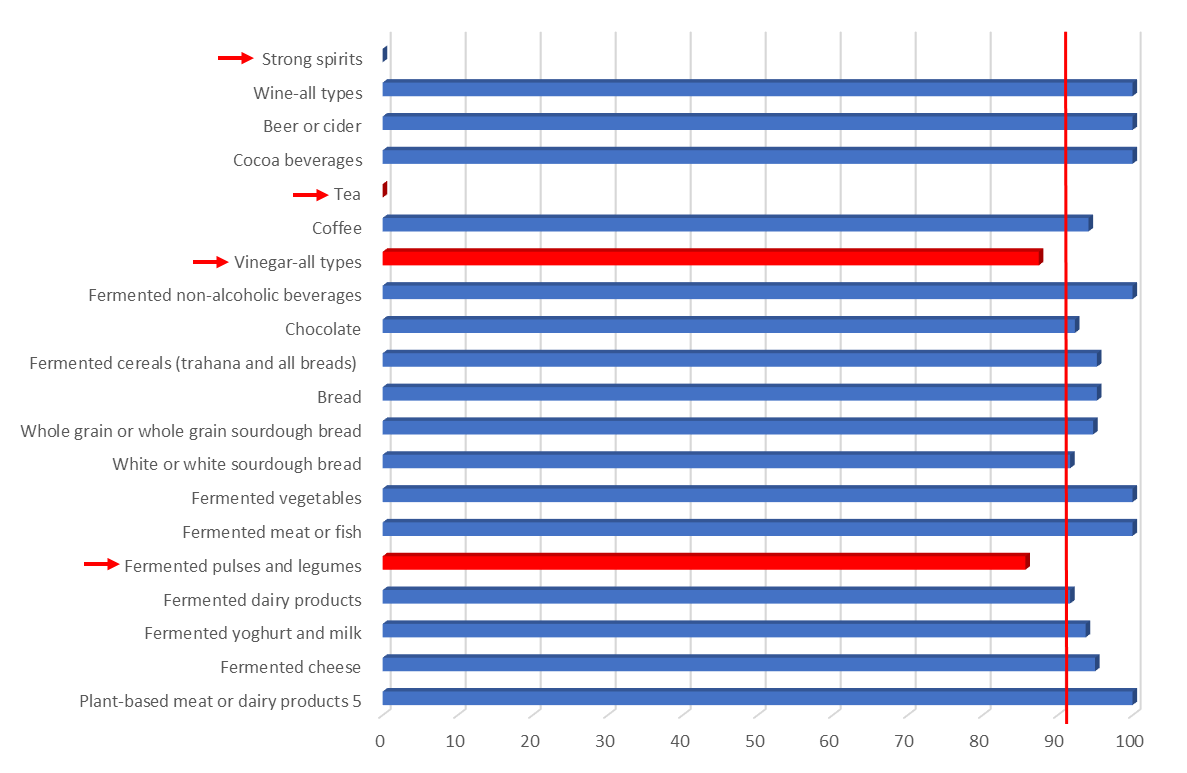


Supplementary Figure S 3. % within the agreement interval to assess the validity of the 3FQ^1,2,3^ for Western Europe *Correlation is significant at the 0.05 level (2-tailed), **Correlation is significant at the 0.001 level (2-tailed).1 3FQ: Fermented Food Frequency Questionnaire.2 n=391 (validity study sample population).3 % within the agreement interval between the results of the 3FQ and the 24hR per food group. Bland and Altman analysis was used to determine the percentage of agreement between the two methods. Red bars indicate the food subcategories that an agreement was not obtained through Bland-Altman analysis.


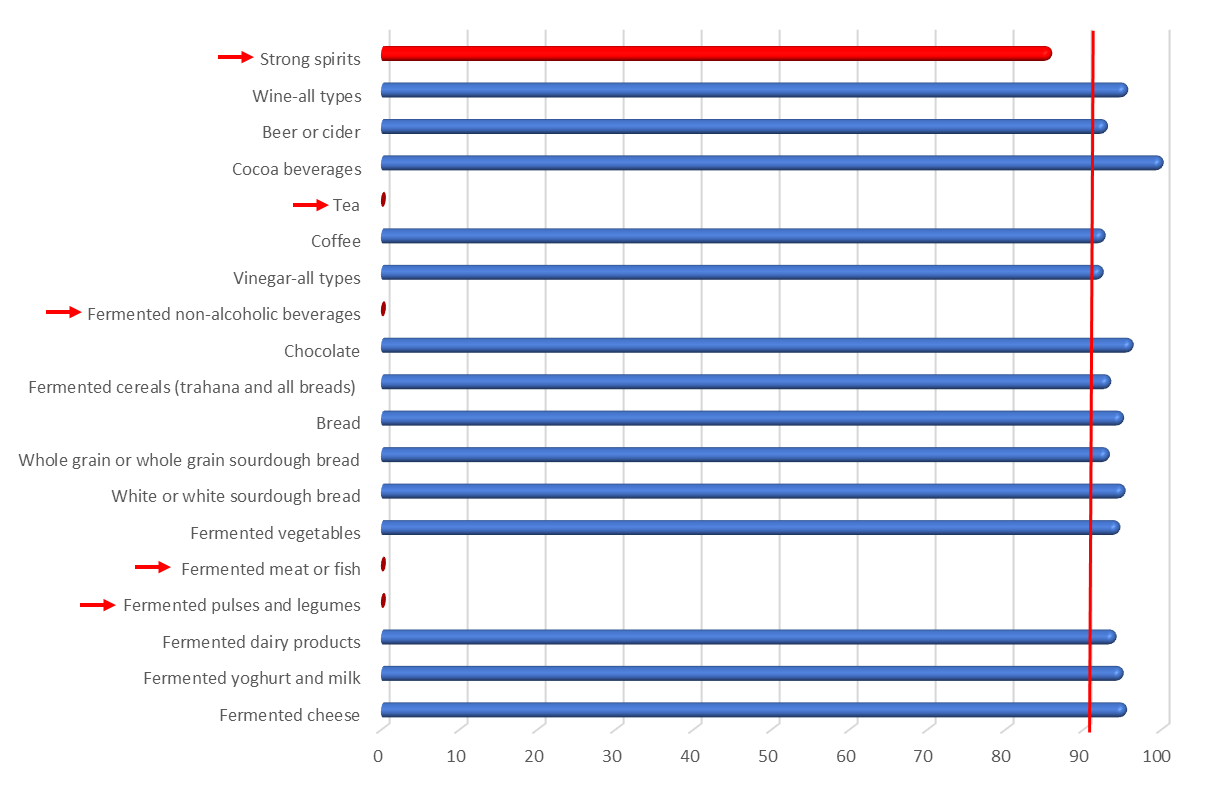


Supplementary Figure S 4. % within the agreement interval to assess the validity of the 3FQ^1,2,3^ for Southern Europe *Correlation is significant at the 0.05 level (2-tailed), **Correlation is significant at the 0.001 level (2-tailed).1 3FQ: Fermented Food Frequency Questionnaire.2 n=391 (validity study sample population).3 % within the agreement interval between the results of the 3FQ and the 24hR per food group. Bland and Altman analysis was used to determine the percentage of agreement between the two methods. Red bars indicate the food subcategories that an agreement was not obtained through Bland-Altman analysis.
